# Quantum topology in the ultrastrong coupling regime

**DOI:** 10.1038/s41598-022-15735-0

**Published:** 2022-07-08

**Authors:** C. A. Downing, A. J. Toghill

**Affiliations:** Department of Physics and Astronomy, University of Exeter, Exeter, EX4 4QL UK

**Keywords:** Quantum physics, Optical physics

## Abstract

The coupling between two or more objects can generally be categorized as strong or weak. In cavity quantum electrodynamics for example, when the coupling strength is larger than the loss rate the coupling is termed strong, and otherwise it is dubbed weak. Ultrastrong coupling, where the interaction energy is of the same order of magnitude as the bare energies of the uncoupled objects, presents a new paradigm for quantum physics and beyond. As a consequence profound changes to well established phenomena occur, for instance the ground state in an ultrastrongly coupled system is not empty but hosts virtual excitations due to the existence of processes which do not conserve the total number of excitations. The implications of ultrastrong coupling for quantum topological systems, where the number of excitations are typically conserved, remain largely unknown despite the great utility of topological matter. Here we reveal how the delicate interplay between ultrastrong coupling and topological states manifests in a one-dimensional array. We study theoretically a dimerized chain of two-level systems within the ultrastrong coupling regime, where the combined saturation and counter-rotating terms in the Hamiltonian are shown to play pivotal roles in the rich, multi-excitation effective bandstructure. In particular, we uncover unusual topological edge states, we introduce a flavour of topological state which we call an anti-edge state, and we reveal the remarkable geometric-dependent renormalizations of the quantum vaccum. Taken together, our results provide a route map for experimentalists to characterize and explore a prototypical system in the emerging field of ultrastrong quantum topology.

## Introduction

The beauty of topology continues to fascinate scientists from an increasing diversity of fields, including more recently the quantum light and matter community^1^. The versatility of modern systems from the nanophotonic^2–4^ to the magnonic^5^ to the ultracold atomic^6^ has significantly enrichened contemporary topological physics. Indeed, quantum topology is advancing at an impressive rate both from a fundamental point of view, including the creation of topological sources of quantum light^7^ and biphoton states^8^, and from the perspective of applications, for example the recent development of topological lasers^9^ and chiral quantum optical devices^10^.

**Figure 1:**
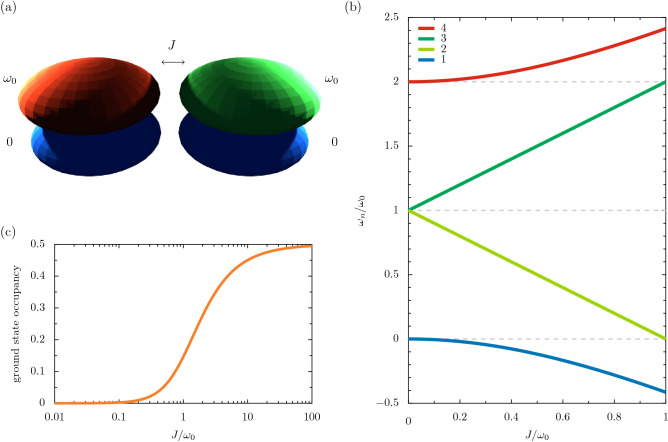
Ultrastrong coupling in the dimer. Panel (**a**): a sketch of a pair of two-level systems, each of bare transition frequency $\omega _0$, with the qubit-qubit coupling strength *J* [cf. Eq. (1)]. Panel (**b**): the eigenfrequencies $\omega _n$, as a function of *J* [cf. Eq. (2)]. Dashed grey lines: the transition frequencies in the uncoupled limit. Panel (**c**): the occupancy of the ground state $||\psi _1\rangle \rangle$ as a function of *J* [cf. Eq. (3d)].

A standard approximation employed within quantum physics is the so-called rotating wave approximation (RWA), whereby all non-resonant terms in the Hamiltonian are discarded as negligible^11^. This celebrated approximation breaks down in the ultrastrong coupling regime^12–14^, where the coupling strength becomes comparable to the bare transition frequencies of the uncoupled subsystems. The coupling is no longer perturbative, such that the non-resonant counter-rotating (C-R) terms must be included in any proper treatment^15–20^. Importantly, these C-R terms permit processes which violate the conservation of the number of excitations, so that the intuitively empty ground state instead bubbles with virtual excitations. The impact of ultrastrong coupling on quantum topological matter^21,22^, which usually draws upon a single particle picture, presents an interesting puzzle.

Ultrastrong coupling has been observed in a stream of pioneering experiments across several platforms, from *LC* resonators magnetically coupled to superconducting qubits^23^, to superconducting artificial atoms coupled to on-chip cavities^24^, to superconducting artificial atoms coupled to the electromagnetic continuum of a one-dimensional waveguide^25^, to arrays of plasmonic nanoparticles^26^. Therefore, an expansion of the field to include topological considerations - so-called ultrastrong quantum topology - is a natural progression which promises a myriad of theoretical and experimental curiosities. Early theoretical efforts have thus far focussed on the influence of the geometric phase and other topological features of the quantum Rabi model^27,28^.

A prototypical model showcasing ultrastrong coupling is a pair of coupled two level systems (2LSs). As sketched in Fig. 1a, we consider the transition frequency of each 2LS to be $\omega _0$, and the coupling strength to be $J > 0$. The Hamiltonian $\hat{H}$, including the C-R terms, thus reads (after setting $\hbar = 1$)^11,29^

1 $$\begin{aligned} \hat{H} = \omega _0 \left( \sigma _1^{\dagger } \sigma _1 + \sigma _2^{\dagger } \sigma _2 \right) + J \left( \sigma _1 + \sigma _1^{\dagger } \right) \left( \sigma _{2} + \sigma _{2}^{\dagger } \right) , \end{aligned}$$

with the raising (lowering) operator $\sigma _n^\dagger$ ($\sigma _n$) describing the *n*-th 2LS. Within the RWA, the terms $\propto \sigma _1 \sigma _2$ and $\propto \sigma _1^{\dagger } \sigma _2^{\dagger }$ in Eq. (1) are discarded and the four resulting eigenfrequencies $\omega _n'$ are simply: $\omega _4' = 2\omega _0$; the hybridized levels $\omega _3' = \omega _0+J$ and $\omega _2' = \omega _0-J$; and the ground state $\omega _1' = 0$. In terms of the bare states in the occupation number representation, that is $|0, 0\rangle$, $|1, 0\rangle$, $|0, 1\rangle$, and $|1, 1\rangle$, one may find the corresponding eigenstates $|\psi _n\rangle$. The extremities of the energy ladder are associated with the doubly occupied eigenstate, which we denote $|\psi _4\rangle = |1, 1\rangle$, and the wholly unoccupied ground state, which we label $|\psi _1\rangle = |0, 0\rangle$. The intermediate, singly-occupied eigenstates $|\psi _3\rangle = \left( |1, 0\rangle + |0, 1\rangle \right) /\sqrt{2}$ and $|\psi _2\rangle = \left( |1, 0\rangle - |0, 1\rangle \right) /\sqrt{2}$ are superpositions due to the nonzero coupling, and are separated in energy by the splitting 2*J*^30,31^.

When considering the ultrastrong coupling regime, diagonalizing the full Hamiltonian of Eq. (1) leads to the exact eigenfrequencies $\omega _n$, which are given by^32^

2a $$\begin{aligned} \omega _{4}&= \omega _0 + \sqrt{ \omega _0^2 + J^2}. \end{aligned}$$

2b $$\begin{aligned} \omega _{3}&= \omega _0 + J, \end{aligned}$$

2c $$\begin{aligned} \omega _{2}&= \omega _0 - J, \end{aligned}$$

2d $$\begin{aligned} \omega _{1}&= \omega _0 - \sqrt{ \omega _0^2 + J^2}. \end{aligned}$$

Notably, the C-R terms $\propto \sigma _1 \sigma _2$ and $\propto \sigma _1^{\dagger } \sigma _2^{\dagger }$ in Eq. (1) link the zero-excitation and two-excitation sectors, breaking particle number conservation. Consequently, the highest and lowest rungs of the energy ladder are renormalized from $\omega _4' = 2\omega _0$ and $\omega _1' = 0$ to the *J*-dependent eigenfrequencies $\omega _4$ and $\omega _1$ respectively [cf. Eq. (2a) and Eq. (2d)]. In Fig. 1b, we plot the four eigenfrequencies $\omega _n$ of Eq. (2) as a function of the coupling strength *J*, showcasing the drastic reconstruction of half of the energy ladder in the ultrastrong coupling regime. In particular, there are divergences from the bare transition frequencies 0 and $2\omega _0$, as marked by the dashed grey lines. The exact eigenstates associated with Eq. (2), where we use the notation $||\psi _n\rangle \rangle$ for when the C-R terms are considered, read

3a $$\begin{aligned} ||\psi _4\rangle \rangle&= \frac{1}{\sqrt{ \omega _{1}^2 + J^2 }} \left( J |1, 1\rangle - \omega _{1} |0, 0\rangle \right) , \end{aligned}$$

3b $$\begin{aligned} ||\psi _3\rangle \rangle&= \frac{1}{\sqrt{2}} \left( |1, 0\rangle + |0, 1\rangle \right) , \end{aligned}$$

3c $$\begin{aligned} ||\psi _2\rangle \rangle&= \frac{1}{\sqrt{2}} \left( |1, 0\rangle - |0, 1\rangle \right) , \end{aligned}$$

3d $$\begin{aligned} ||\psi _1\rangle \rangle&= \frac{1}{\sqrt{ \omega _{4}^2 + J^2 }} \left( \omega _{4} |0, 0\rangle - J |1, 1\rangle \right) , \end{aligned}$$

where the frequencies $\omega _{1}$ and $\omega _{4}$ are defined in Eq. (2). Clearly, the mixed particle number eigenstates of Eq. (3a) and Eq. (3d) present a new paradigm for coupled systems. One immediate implication is that the ground state $||\psi _1\rangle \rangle$ is no longer trivially empty, $||\psi _1\rangle \rangle \ne |0, 0\rangle$. As shown in Fig. 1c, the occupancy of the proper ground state $||\psi _1\rangle \rangle$ increases with coupling strength *J*. When $J \ll \omega _0$, the pure state $||\psi _1\rangle \rangle \simeq |0, 0\rangle$ means that the ground state is wholly unoccupied, while in the extreme limiting case of $J \gg \omega _0$ the maximally mixed state of $||\psi _1\rangle \rangle \simeq \left( |0, 0\rangle - |1, 1\rangle \right) /\sqrt{2}$ is essentially reached, such that the ground state $||\psi _1\rangle \rangle$ is half-occupied and half-unoccupied.

**Figure 2:**
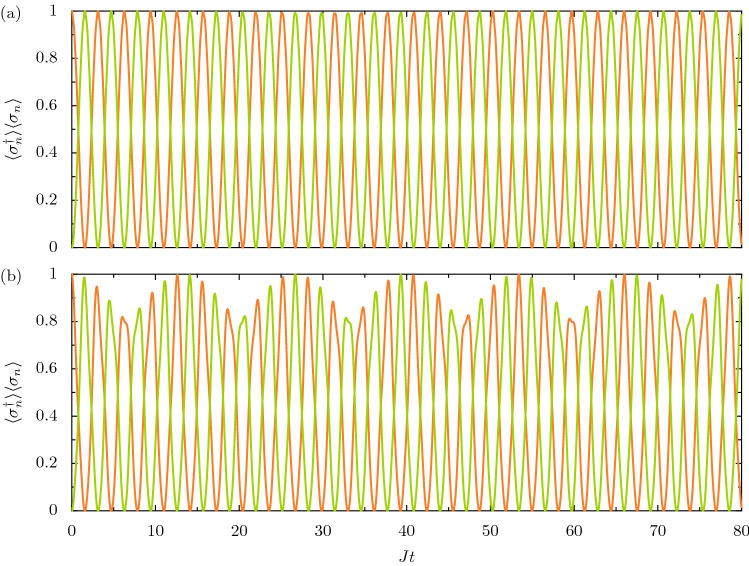
Mean correlations in the dimer. The evolution of $\langle \sigma _n^\dagger \rangle \langle \sigma _n \rangle$ as a function of time *t* for the *n*-th 2LS, in units of the inverse coupling strength $J^{-1}$ [cf. Eq. (4) with Eq. (5)]. Orange lines: $n = 1$. Green lines: $n = 2$. Panel (**a**): the strong coupling regime, where the bare transition frequency $\omega _0 = 10 J$. Panel (**b**): the ultrastrong coupling regime, where $\omega _0 = 2 J$.

The impact of the ultrastrong coupling can also be seen in the mean correlations of the dimer. For example, the population-like quantity $\langle \sigma _n^\dagger \rangle \langle \sigma _n \rangle$ reads (see the Supplementary Information S1)

4a $$\begin{aligned} \langle \sigma _1^\dagger \rangle \langle \sigma _1 \rangle&= f\left( \tilde{\omega }_0, t \right) \, \cos ^2 \left( J t \right) , \end{aligned}$$

4b $$\begin{aligned} \langle \sigma _2^\dagger \rangle \langle \sigma _2 \rangle&= f\left( \tilde{\omega }_0, t \right) \, \sin ^2 \left( J t \right) , \end{aligned}$$

where the auxiliary function $f\left( \tilde{\omega }_0, t \right)$, which accounts for the ultrastrong coupling, is given by

5 $$\begin{aligned} f\left( \tilde{\omega }_0, t \right) = \cos ^2 \left( \tilde{\omega }_0 t \right) + \left( \frac{\omega _0}{\tilde{\omega }_0} \right) ^2 \sin ^2 \left( \tilde{\omega }_0 t \right) , \end{aligned}$$

with the renormalized transistion frequency $\tilde{\omega }_0 = \sqrt{ \omega _0^2 + J^2}$. In the strong coupling limit of $J \ll \omega _0$, the function $f\left( \tilde{\omega }_0, t \right) \simeq 1$ and Eq. (4) approaches the simple trigonometric results $\langle \sigma _1^\dagger \rangle \langle \sigma _1 \rangle \simeq \cos ^2 \left( J t \right)$ and $\langle \sigma _2^\dagger \rangle \langle \sigma _2 \rangle \simeq \sin ^2 \left( J t \right)$, which recover the expressions found after neglecting the C-R terms in the Hamiltonian of Eq. (1). Otherwise, Eq. (4) presents nontrivalities. In Fig. 2 the evolution of the population-like cycles of $\langle \sigma _n^\dagger \rangle \langle \sigma _n \rangle$ are displayed as a function of time *t* using Eq. (4), where the orange and green lines represent the first and second 2LS respectively [cf. Fig. 1a]. Panel (a), where $J = \omega _0/10$, shows the typical strong coupling regime result: periodic cycles of essentially unity amplitude, which are well described by trigonometric functions in the dimensionless quantity *Jt*. The ultrastrong coupling regime $\omega _0 \sim J$ sees the renormalized transition frequency $\tilde{\omega }_0$ influence the amplitude of the cycles following Eq. (5). This is typified by panel (b), where $J = \omega _0/2$ and a characteristic wave packet is observed oscillating in time. The C-R terms also also impact the entanglement properties of the dimer, as discussed in the Supplementary Information S1.

The consequences of ultrastrong coupling for quantum topology is mostly unchartered terrain. We will start to explore it via the celebrated Su-Schrieffer-Heeger (SSH) topological array model^6,21,33^, which is formed by a chain of dimers like the one described in Fig. 1.

**Figure 3:**
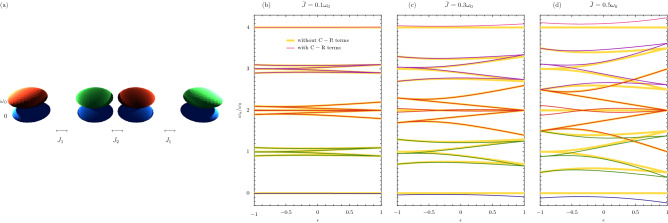
Ultrastrong coupling in the dimerized chain. Panel (**a**): a sketch of a bipartite array pair of two-level systems, each of bare transition frequency $\omega _0$, with alternating coupling strengths $J_1$ and $J_2$ along the chain of size $N = 4$ [cf. Eq. (6)]. Panels (**b**–**d**): the eigenfrequencies as a function of the dimerization parameter $\epsilon$, for increasing coupling strength $\bar{J}$ across the panels [cf. Eq. (7)]. Thin colored lines: the eigenfrequencies $\omega _n$, found by diagonalizing the full Hamiltonian of Eq. (6). Thick yellow lines: $\omega _n^\prime$, found upon neglecting the C-R terms.

## Results

Let us consider a dimerized chain of 2LSs, each of bare transition frequency $\omega _0$, with alternating coupling strengths $J_1 > 0$ and $J_2 > 0$ along the formed one-dimensional lattice^21^. The arrangement is sketched in Fig. 3a for a short example chain of four 2LSs. For a general chain of size *N*, the Hamiltonian $\hat{H}$ reads [cf. Eq. (1)]

6 $$\begin{aligned} \hat{H} = \omega _0 \sum _{n=1}^{N} \sigma _n^{\dagger } \sigma _n + J_1 \sum _{n=1}^{\lfloor {\frac{N}{2}}\rfloor } \left( \sigma _{2n} + \sigma _{2n}^{\dagger } \right) \left( \sigma _{2n-1} + \sigma _{2n-1}^{\dagger } \right) + J_2 \sum _{n=1}^{\lfloor {\frac{N-1}{2}}\rfloor } \left( \sigma _{2n} + \sigma _{2n}^{\dagger } \right) \left( \sigma _{2n+1} + \sigma _{2n+1}^{\dagger } \right) , \end{aligned}$$

where the floor function $\lfloor {...}\rfloor$ ensures Eq. (6) holds for both even and odd values of the integer *N*. The model encapsulated by Eq. (6) has an equivalent $2^N \times 2^N$ matrix representation, and in the RWA there are well-defined sectors corresponding to the conserved number of excitations $\mathcal {N}$. The sectors follow Pascal’s triangle, where the binomial coefficient $N!/ \mathcal {N}!/(N-\mathcal {N})!$ counts the number of eigenstates in each excitation sector $\mathcal {N}$ (see the Supplementary Information S1). For a chain of $N = 4$ 2LSs, the $2^4 = 16$-dimensional Hilbert space is distributed with $\{1, 4, 6, 4, 1\}$ eigenstates in the $\mathcal {N} = \{ 0, 1, 2, 3, 4\}$ excitation sectors respectively (when working in the RWA). That is, there is a single excitation-less ($\mathcal {N} = 0$) ground state, four single-excitation ($\mathcal {N} = 1$) states, six two-excitation ($\mathcal {N} = 2$) states and so on. Two principal parameters govern the intrinsic physics of the model defined by Eq. (6),

7 $$\begin{aligned} \epsilon = \frac{J_1-J_2}{\bar{J}}, \quad \quad \quad \quad \quad \quad \quad \quad \quad \quad \quad \bar{J} = J_1 + J_2, \end{aligned}$$

namely $\epsilon$, the dimerization parameter which records the inherent geometry of the bipartite chain; and $\bar{J}$, the chain coupling strength. The latter quantity tracks the importance of the C-R terms like $\propto \sigma _l \sigma _m$ and $\propto \sigma _l^{\dagger } \sigma _m^{\dagger }$ in Eq. (6) via the dimensionless ratio $\bar{J}/\omega _0$. The inverse relations for Eq. (7) provide forms of the alternating coupling strengths $J_1 = \left( 1 + \epsilon \right) \bar{J} / 2$ and $J_2 = \left( 1 - \epsilon \right) \bar{J} / 2$.

**Figure 4:**
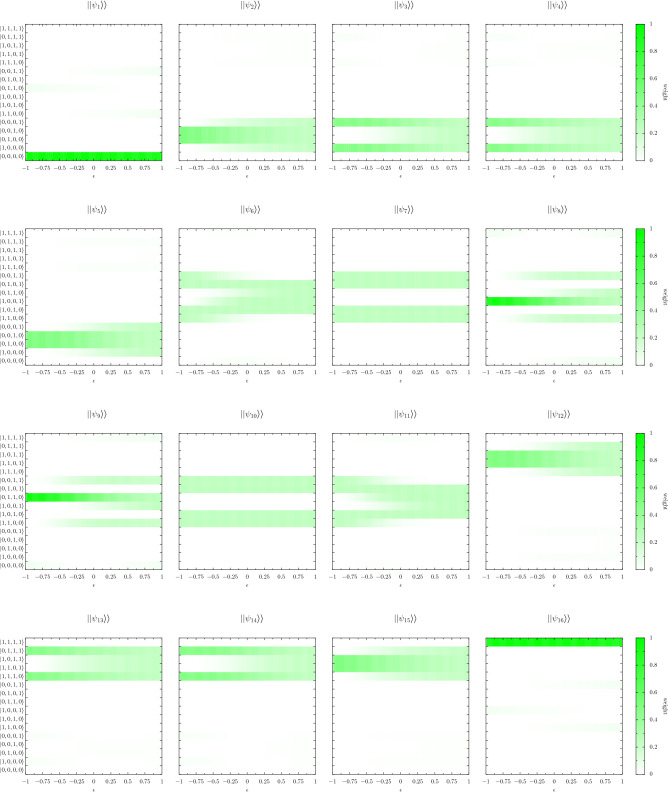
Eigenstates in the dimerized chain. The probability densities of the eigenstates $||\psi _n\rangle \rangle$ in the ultrastrong coupling regime, in terms of the bare states $|i, j, k, l\rangle$, as a function of the dimerization $\epsilon$ [cf. Eq. (7)]. In the figure, the coupling strength $\bar{J} = 0.5 \omega _0$, and the chain is of size $N = 4$ (corresponding to a $2^4=16$ dimensional Hilbert space). The presented eigenstates correspond to the eigenfrequencies of Fig. 3d.

**Figure 5:**
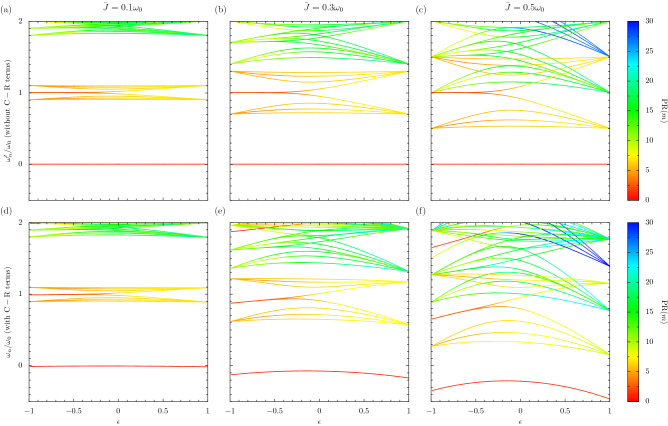
Topology in the ultrastrong coupling regime. Eigenfrequencies as a function of the dimerization parameter $\epsilon$, with increasing coupling strength $\bar{J}$ in each column [cf. Eq. (7)]. Colour bars: the participation ratio $\mathrm {PR}(n)$ of each eigenstate. Panels (**a**–**c**): the results without taking counter-rotating terms in the Hamiltonian of Eq. (6) into account, namely the RWA eigenfrequencies $\omega _n^\prime$ and RWA eigenstates $|\psi _n\rangle$. Panels (**d**–**f**): the full eigenfrequencies $\omega _n$ and eigenstates $||\psi _n\rangle \rangle$ are used. In the figure, the chain is of size $N = 8$ (corresponding to a $2^8=256$ dimensional Hilbert space) and all data above $2\omega _0$ is cut.

The results of diagonalizing Eq. (6) are shown in Fig. 3b–d for a chain of $N = 4$ 2LSs, which is represented in Fig. 3a. The panels (b–d) display the eigenfrequencies as a function of the dimerization $\epsilon$, with increasing coupling strength $\bar{J}$ across the panels. In panel (b) the chain coupling strength $\bar{J} = 0.1 \omega _0$, such that the 16 exact eigenfrequencies $\omega _n$ (thin colored lines) closely resemble the results in the RWA (thick yellow lines). Notably, the effective bandstructure with dimerization $\epsilon < 0$ is markedly different from that with $\epsilon > 0$, especially around $\omega _0$ and $3\omega _0$ where states are either present or absent depending upon the dimerization (hinting at the topology of the model). Upon increasing the coupling strength to $\bar{J} = 0.3 \omega _0$ in panel (c), the influence of the C-R terms becomes visible, particularly around 0 and $4\omega _0$, where there are significant deviations from the RWA results [as may have been anticipated from the dimer results of Fig. 1b, where the 0 and 2 excitation sectors became linked]. Finally, in panel (d) $\bar{J} = 0.5 \omega _0$, such that the impact of entering the ultrastrong coupling regime is highly apparent. It has lead even to crossovers between energy levels supposedly (in the RWA) associated with different numbers of excitations. For example, green (nominally single excitation sector) and red (supposedly two excitation sector) eigenfrequencies overlap when $\epsilon > 0$, as do the red and purple (purportedly three excitation sector) results. However, while there is a significant reconstruction of the energy ladder, the asymmetry about $\epsilon = 0$ in panel (d) remains in a similar fashion to panel (a), hinting that some topological properties may well endure.

The underlying behavior of the eigenstates associated with Fig. 3d is investigated in Fig. 4, where we consider the fidelity of the 16 quantum states from $||\psi _1\rangle \rangle$ to $||\psi _{16}\rangle \rangle$, ordered from lowest to highest in energy (from $\omega _1$ to $\omega _{16}$). That is, we show how the weighting of the underlying bare states $|i, j, k, l\rangle$ changes as a function of the dimerization $\epsilon$, with white signifying zero contribution from the bare state and brighter shades of green denoting an increasingly large overlap (the bare states $|i, j, k, l\rangle$ are marked at the left edge of the first column of panels). The eigenstates labelled $||\psi _3\rangle \rangle$ and $||\psi _4\rangle \rangle$ correspond to the states residing near to $\omega _0$ (when $\epsilon < 0$) in Fig. 3d. The overlaps of $||\psi _3\rangle \rangle$ and $||\psi _4\rangle \rangle$ (found at the right-hand side of the upper row of Fig. 4) demonstrates their transition from being primarily edge states when $\epsilon < 0$, composed of the bare states $|1, 0, 0, 0\rangle$ and $|0, 0, 0, 1\rangle$, to being bulk states extended throughout the four-dimensional single excitation sector when $\epsilon > 0$. An analogous effect may be observed for the eigenstates $||\psi _{13}\rangle \rangle$ and $||\psi _{14}\rangle \rangle$, which reside around $3\omega _0$ (when $\epsilon < 0$) in Fig. 3d. Now it is a ‘hole’, or absence of excitation, which appears as a novel type of topological anti-edge state, based upon the bare states $|1, 1, 1, 0\rangle$ and $|0, 1, 1, 1\rangle$ (see the two panels on the left-hand side of the bottom row of Fig. 4). These edge state and anti-edge state features are common across chains of an arbitrary size *N*, even in the ultrastrong coupling regime (see the Supplementary Information S1). Indeed, despite the significant coupling strength of $\bar{J} = 0.5 \omega _0$ the packaging of states into excitation-number-conserving bundles is still has some utility. The influence of the C-R terms on the eigenstates $||\psi _n\rangle \rangle$ can be seen for example in the bottom right panel of Fig. 4, describing the eigenstate $||\psi _{16}\rangle \rangle$, where the deviation from the RWA result of $|1, 1, 1, 1\rangle$ arises in the noticeable contributions of the two-excitation sector bare states.

The topology of a dimerized chain of 2LSs in the ultrastrong coupling regime is charted in Fig. 5 for a typical non-short chain. There we plot the eigenfrequencies as a function of the dimerization parameter $\epsilon$, with increasing coupling strength $\bar{J}$ for each column. The top row employs the RWA and as such is applicable within strong coupling, while the bottom row takes the C-R terms into account so that ultrastrong coupling may also be properly described. In the figure, the chain is of size $N = 8$ (corresponding to a $2^8=256$-dimensional Hilbert space) and all data above $2\omega _0$ is cut. The color bar measures the localization of each state via $\mathrm {PR}(n)$, the participation ratio^34–37^ of each eigenstate. In the participation ratio calculation, $|\psi _{n}\rangle$ is used in the upper row and $||\psi _{n}\rangle \rangle$ in the lower row. This localization measure counts how many bare states contribute to the eigenstate, with red marking a low number of states (a signature of potential edge states), and blue a high number of states (a signifier of highly extended states spread out over the entire chain).

In first column of Fig. 5, where the coupling strength $\bar{J} = 0.1 \omega _0$ effectively ensures strong coupling behavior, the results of panels (a) and (d) are in essence the same. One observes in red the topological edge state at $\omega _0$ for $\epsilon < 0$, which lies in the effective band gap between two collections of extended states in orange-yellow. This midgap state disappears when $\epsilon > 0$, a result resembling standard one-excitation SSH-like models transitioning from the topologically nontrivial to the topologically trivial regime^21^. The lowest energy state $\omega _1^\prime = 0$ in panel (a) [or $\omega _1 \simeq 0$ in panel (d)] is not topological, and it is red simply because it is only composed of the empty state $|0, 0, 0, 0\rangle$ [in panel (d), $||\psi _1\rangle \rangle \simeq |0, 0, 0, 0\rangle$ holds].

The second column of Fig. 5 sees the coupling strength increased to $\bar{J} = 0.3 \omega _0$. Similar topological features can be found around $\omega _0$, with the red localized state present when $\epsilon < 0$ merging into the effective bands for $\epsilon > 0$. However, now the two effective bands surrounding the edge state are of substantially larger effective bandwidths due to the increased coupling strength $\bar{J}$, such that the nominally two-excitation-sector states in the vicinity of $2\omega _0$ (primarily colored in green) are almost reaching the ostensible one-excitation-sector states around $\omega _0$ (mostly orange-yellow). While the orange-yellow extended states in panel (b) maintain symmetric behavior about $\omega _0$ due to the RWA, the analogous collections of states in panel (d) are highly asymmetric, since the C-R terms guarantee significant couplings to higher rungs of the energy ladder. Most notably, these hybridizations lead to a ground state no longer pinned at 0 in panel (d).

When $\bar{J} = 0.5 \omega _0$ in the third column of Fig. 5, there is a drastic reconstruction of the effective bandstructure in panel (f) due to the ultrastrong coupling causing a complete breakdown of particle number conservation. The ground state shows a remarkable warping away from 0, and there is pronounced effective band crossing around $\omega _0$ for all values of $\epsilon$. However, quite remarkably, the edge state (now located near to $0.7 \omega _0$ when $\epsilon \simeq -1$) persists, as does its highly localized nature (it is red for $\epsilon < 0$ and indeed transforms into an extended state in yellow-green for $\epsilon > 0$) suggesting the translation of topological features deep into the ultrastrong coupling regime. This relative robustness to the effects of the C-R terms in the Hamiltonian presents intriguing perspectives for the existence of localized edge states specifically, and for ultrastrong quantum topology in general.

**Figure 6:**
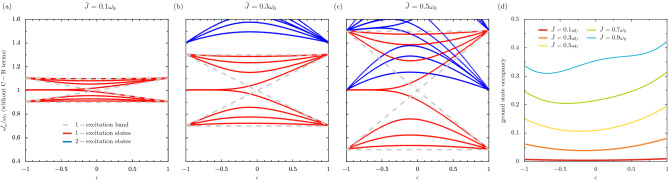
Effective band theory and renormalization of the vacuum. Panels (**a**–**c**): the RWA eigenfrequencies for a finite chain $\omega _n^\prime$ as a function of the dimerization parameter $\epsilon$, with increasing coupling strength $\bar{J}$ in each column [cf. Eq. (7)]. Dashed grey lines: the boundaries of the RWA eigenfrequencies in the one-excitation sector, as found from a periodic calculation in the continuum limit $N \rightarrow \infty$ [cf. Eq. (8)]. Solid lines: one-excitation states are shown in red and two-excitation states are shown in blue. All data above $1.6\omega _0$ and below $0.4\omega _0$ is cut. Panel (**d**): The occupancy of the ground state of the dimerized chain as a function of $\epsilon$, where the $\bar{J}$ increases with increasing line thickness. In the figure, $N = 8$.

The preceding discussion of Fig. 5 panels (a–f) used terminology such as effective bandstructures, effective bands and effective band gaps. Such concepts may reasonably be invoked, since the finite system studied using the Hamiltonian of Eq. (6) has a natural analogue in the continuum limit. When the number of 2LSs in the chain $N \rightarrow \infty$, the resultant eigenfrequencies may be grouped into certain bands which are separated by certain band gaps. For example, applying the RWA and concentrating on the first-excitation sector only, Eq. (6) may be readily diagonalized as $\omega _0 \pm \sqrt{J_1^2 + J_2^2 + 2 J_1 J_2 \cos \left( q d\right) }$, where *q* is the wavenumber and where periodic boundary conditions were employed to ensure an analytic result^21^. This simple expression exposes the presence of two bands (± in the aforementioned expression) filled of one-excitation states, which appear inside the bow tie shape formed by

8 $$\begin{aligned} \omega _0 + \bar{J}, \quad \quad \quad \quad \quad \quad \omega _0 + |\epsilon | \bar{J}, \quad \quad \quad \quad \quad \quad \omega _0 - |\epsilon | \bar{J}, \quad \quad \quad \quad \quad \quad \omega _0 - \bar{J}, \end{aligned}$$

which are plotted as the dashed grey lines in Fig. 6a–c. We also plot the RWA results for a finite chain of size $N = 8$ in panels (a, b, c), for increasing values of the coupling strength $\bar{J}$, which clearly fit the bow tie analysis of Eq. (8) for the one-excitation eigenfrequencies (red lines). The two-excitation eigenfrequencies (blue lines) encroaching the bow tie for larger $\bar{J}$ also form a band, but no simple analytic expression for the two-excitation band is readily obtainable. Similar results to Fig. 6a–c also hold with the C-R terms included, but since the number of excitations is no longer conserved the effective band theory language becomes less and less useful for larger values of $\bar{J}$. Notably, the edge states falling outside of the bow ties in Fig. 6a–c are a priori excluded from the periodic boundary condition calculation leading to Eq. (8).

As was mentioned in the discussion of a 2LS dimer around Fig. 1c, ultrastrong coupling renormalizes the ground state due to the presence of the C-R terms in the Hamiltonian of Eq. (6) linking the vacuum state and occupied states. This effect can be clearly seen for the dimerized chain by considering the occupancy of the ground state $||\psi _1\rangle \rangle$, as is shown in Fig. 6d as a function of the dimerization $\epsilon$. The coupling strength $\bar{J}$ increases from $0.1\omega _0$ to $0.9\omega _0$ with decreasing line thickness. Quite intuitively, with smaller $\bar{J}$ (thick red line) the ground state is essentially unoccupied $||\psi _1\rangle \rangle = |0, 0, \ldots , 0\rangle$, being the trivial ground state familiar from the RWA. However, upon entering ultrastrong coupling (thinner lines), there is a significant occupation of the ground state $||\psi _1\rangle \rangle$. Importantly, this effect is highly sensitive to the dimerization $\epsilon$, and thus may be readily probed by taking measurements of the same physical system in different geometric configurations.

## Discussion

We have studied a prototypical topological model in the ultrastrong coupling regime, where the rotating wave approximation breaks down. Inspired by the surge in experimental activity on one-dimensional dimerized arrays^38–41^, we have shown that in the ultrastrong coupling regime various desirable features remain. For example, topological edge states continue to form in the gaps in the effective bandstructure, and although they are no longer pinned at a constant ‘zero energy’ they are highly localized in the topologically nontrivial geometric arrangement and indeed disappear in the trivial arrangement. Consideration of higher rungs of the energy ladder led to the discovery of anti-edge states, which have the property of residing everywhere apart from the edges of the chain. Finally, a hallmark of ultrastrong coupling is a non-empty ground state, and here we show that this vacuum renormalization is geometric-dependent for a dimerized chain, proffering opportunities for observation. We believe that our results should stimulate experimental work^23–26^ in ultrastrong topology, as well as further theoretical work in this exciting area.

## Methods

In this theoretical work, exact diagonalization of finite matrices was carried out as described in the main text. Further details are given in the Supplementary Information S1.

## Supplementary Information

Supplementary Information.

## Data Availability

There is no additional data. Further information is given in the Supplementary Information.
