# Supplementary material for: Quantum topology in the ultrastrong coupling regime
Source: Sci Rep. 2022 Jul 8;12:11630. doi: 10.1038/s41598-022-15735-0 (PMC9270417; doi:10.1038/s41598-022-15735-0)
Supplement: Supplementary file 1 — Supplementary Information. [file 41598_2022_15735_MOESM1_ESM.pdf]

Supplementary Information:
Quantum topology in the ultrastrong coupling regime

C. A. Downing*[1] and A. J. Toghill[1]

[1](c.a.downing@exeter.ac.uk) *Department of Physics and Astronomy, University of Exeter, Exeter EX4 4QL, United Kingdom*

In this Supplementary Information we provide an in-depth treatment of various models of coupled two-level systems. We build up to the full dimerized chain theory within the ultrastrong coupling regime, which is the subject of the main text. In particular, we provide analytical results describing dimers, trimers, and regular chains of coupled two-level systems beyond the rotating wave approximation.

CONTENTS

I. A pair of coupled two-level systems 2
A. Strong coupling 2
B. Ultrastrong coupling 3
C. Correlations 4
D. Dynamics 6

II. A triple of coupled two-level systems 7
A. Strong coupling 8
B. Ultrastrong coupling 8

III. A quadruple of coupled two-level systems 10
A. Strong coupling 10
B. Ultrastrong coupling 12

IV. A chain of coupled two-level systems 12
A. Strong coupling 13
B. Ultrastrong coupling 13

V. A dimerized triple of coupled two-level systems 14
A. Strong coupling 15
B. Ultrastrong coupling 15

I. A PAIR OF COUPLED TWO-LEVEL SYSTEMS

Let us consider a pair of coupled two-level systems (2LSs), as discussed at the start of the main text. The Hamiltonian reads

$$\hat{H} = \omega_0 \left(\sigma_1^\dagger \sigma_1 + \sigma_2^\dagger \sigma_2 \right) + J \left(\sigma_1 + \sigma_1^\dagger \right) \left(\sigma_2 + \sigma_2^\dagger \right), \tag{S1}$$

where the bare transition frequency is ω_0, and the coupling strength is J. Arranging by the number $\mathcal{N}$ of excitations in the system, the bare state basis is

$$\mathcal{N} = 0 \qquad |0,0\rangle, \tag{S2a}$$
$$\mathcal{N} = 1 \qquad \sigma_1^\dagger |0,0\rangle = |1,0\rangle, \qquad \sigma_2^\dagger |0,0\rangle = |0,1\rangle, \tag{S2b}$$
$$\mathcal{N} = 2 \qquad \sigma_1^\dagger \sigma_2^\dagger |0,0\rangle = |1,1\rangle, \tag{S2c}$$

leading to a representation of Eq. (S1) with the 4×4 matrix

$$H = \begin{pmatrix} 0 & 0 & 0 & J \\ 0 & \omega_0 & J & 0 \\ 0 & J & \omega_0 & 0 \\ J & 0 & 0 & 2\omega_0 \end{pmatrix}. \tag{S3}$$

A. Strong coupling

In the strong coupling regime one may apply the rotating wave approximation (RWA) to Eq. (S1), such that the counter-rotating (C-R) terms $\propto \sigma_1^\dagger \sigma_2^\dagger$ and $\propto \sigma_1 \sigma_2$ are discarded. Then the matrix representation of Eq. (S3) collapses into the block diagonal form

$$H' = \text{diag}\{H_0', H_1', H_2'\}, \tag{S4}$$

where the three contributions (corresponding to the 0, 1, and 2 excitation sectors) are

$$H_0' = 0, \qquad H_1' = \begin{pmatrix} \omega_0 & J \\ J & \omega_0 \end{pmatrix}, \qquad H_2' = 2\omega_0. \tag{S5}$$

The Hamiltonian H' may be diagonalized by Bogoliubov transformation into

$$H' = \omega_1' |\psi_1\rangle\langle\psi_1| + \omega_2' |\psi_2\rangle\langle\psi_2| + \omega_3' |\psi_3\rangle\langle\psi_3| + \omega_4' |\psi_4\rangle\langle\psi_4|, \tag{S6}$$

where the four strong coupling regime eigenfrequencies ω_n' are given by

$$\omega_4' = 2\omega_0. \tag{S7a}$$
$$\omega_3' = \omega_0 + J, \tag{S7b}$$
$$\omega_2' = \omega_0 - J, \tag{S7c}$$
$$\omega_1' = 0. \tag{S7d}$$
$$\tag{S7e}$$

The associated eigenstates $|\psi_n\rangle$ in the strong coupling regime read

$$|\psi_4\rangle = |1,1\rangle, \tag{S8a}$$
$$|\psi_3\rangle = \frac{1}{\sqrt{2}} \left(|1,0\rangle + |0,1\rangle \right), \tag{S8b}$$
$$|\psi_2\rangle = \frac{1}{\sqrt{2}} \left(|1,0\rangle - |0,1\rangle \right), \tag{S8c}$$
$$|\psi_1\rangle = |0,0\rangle. \tag{S8d}$$

Notably, the completely unoccupied ground state $|\psi_1\rangle$ and the doubly-occupied $|\psi_4\rangle$ are both at energies independent of the coupling strength J. The intermediate, singly-occupied states $|\psi_2\rangle$ and $|\psi_3\rangle$ are hybridized in nature, and display a Rabi splitting of $\omega_3' - \omega_2' = 2J$. These features are plotted in Fig. S1 (a), which displays the eigenfrequencies ω_n' as a function of J.

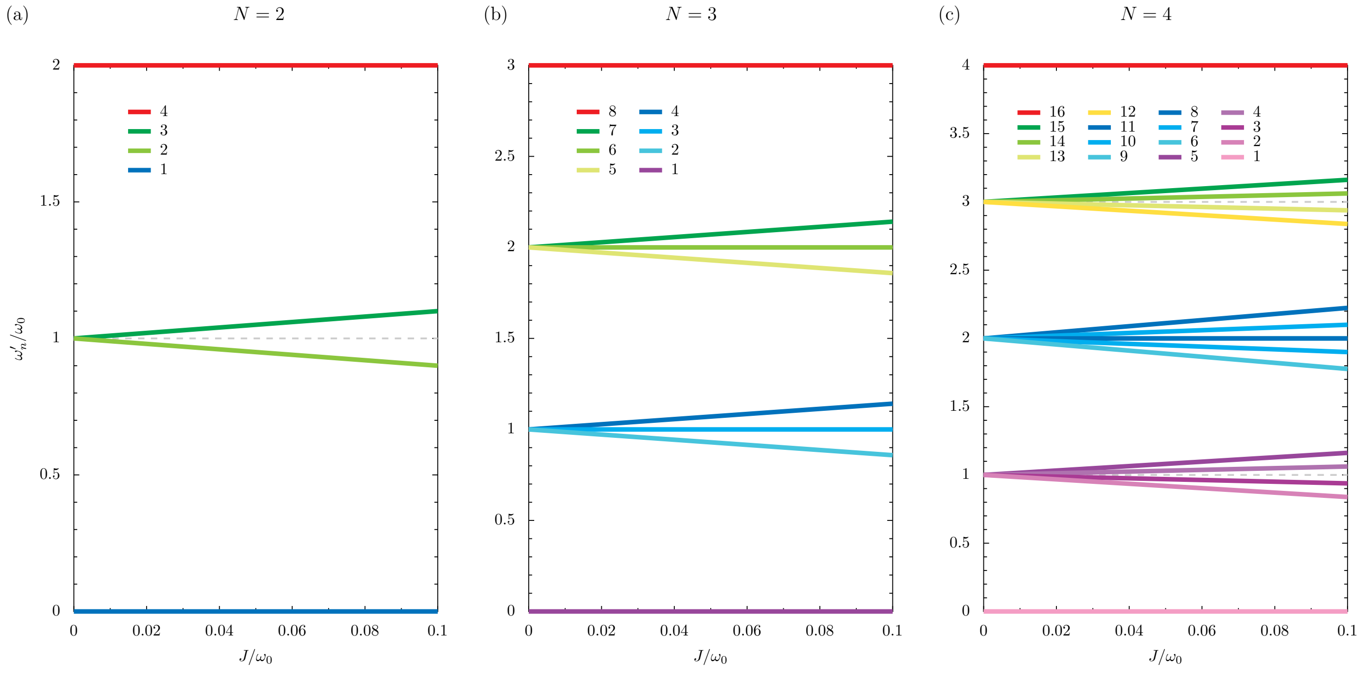

FIG. S1. **Strong coupling eigenfrequencies.** Eigenfrequencies ω'_n in the strong coupling regime, as a function of the coupling strength J (in units of the transition frequency ω_0). We consider a chain of N coupled two-level systems. Panels (a, b, c): the results for a pair [cf. Eq. (S11)], a triple [cf. Eq. (S40)], and a quadruple [cf. Eq. (S49)] of two-level systems respectively ($N = 2, 3, 4$). Dashed grey lines: bare transition frequencies.

B. Ultrastrong coupling

Within the ultrastrong coupling regime the C-R terms in Eq. (S1) are important, so that the governing matrix Hamiltonian is given by Eq. (S3). Since these C-R terms link the 0 and 2 excitation sectors, while leaving the 1 excitation sector unchanged from Eq. (S5), one can rewrite Eq. (S3) as

$$H_{0\leftrightarrow 2} = \begin{pmatrix} 0 & J \\ J & 2\omega_0 \end{pmatrix}, \qquad\qquad H_1 = \begin{pmatrix} \omega_0 & J \\ J & \omega_0 \end{pmatrix}. \tag{S9}$$

Diagonalization of these matrices leads to the transformation of Eq. (S1) into [cf. Eq. (S6)]

$$H = \omega_1 ||\psi_1\rangle\rangle\langle\langle\psi_1|| + \omega_2 ||\psi_2\rangle\rangle\langle\langle\psi_2|| + \omega_3 ||\psi_3\rangle\rangle\langle\langle\psi_3|| + \omega_4 ||\psi_4\rangle\rangle\langle\langle\psi_4||, \tag{S10}$$

where we have used $||\psi_n\rangle\rangle$ to distinguish the exact eigenstates from those only valid in the strong coupling regime. The eigenfrequencies ω_n in the ultrastrong coupling regime read [cf. Eq. (S7)]

$$\omega_4 = \omega_0 + \tilde{\omega}_0. \tag{S11a}$$
$$\omega_3 = \omega_0 + J, \tag{S11b}$$
$$\omega_2 = \omega_0 - J, \tag{S11c}$$
$$\omega_1 = \omega_0 - \tilde{\omega}_0, \tag{S11d}$$

where we have introduced the frequency

$$\tilde{\omega}_0 = \sqrt{\omega_0^2 + J^2}, \tag{S12}$$

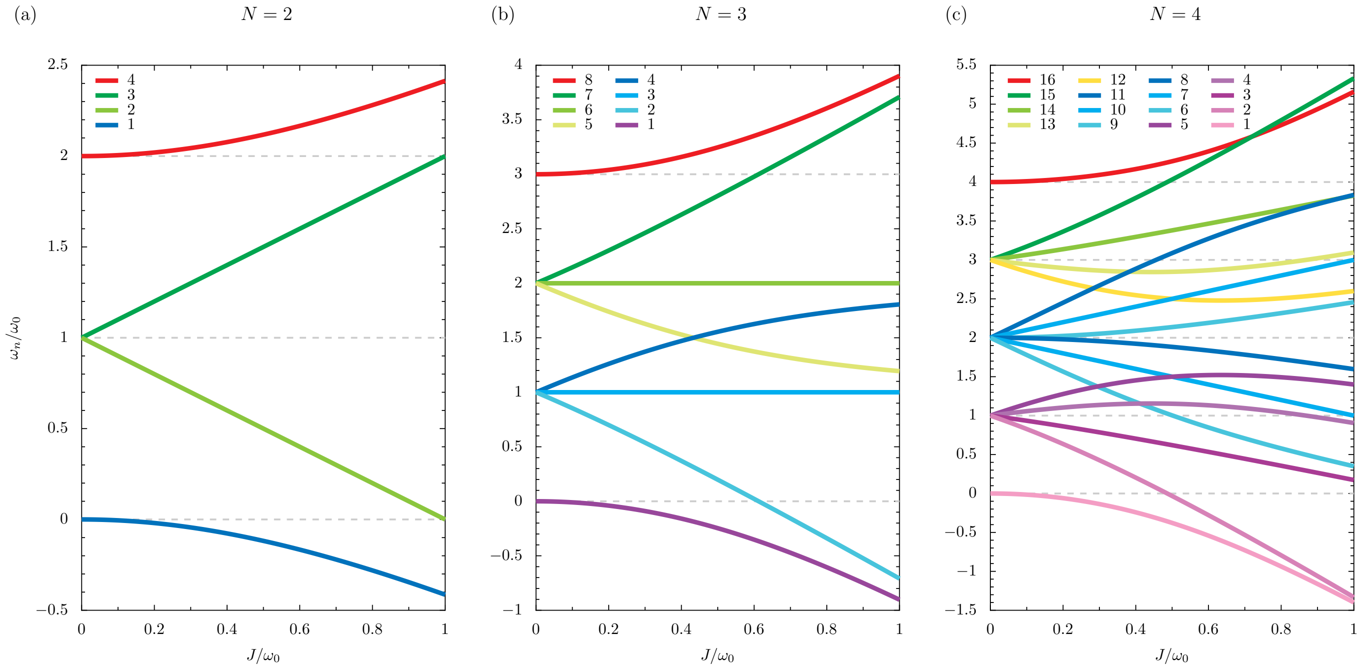

FIG. S2. **Ultrastrong coupling eigenfrequencies.** Eigenfrequencies ω_n in the ultrastrong coupling regime, as a function of the coupling strength J (in units of the transition frequency ω_0). We consider a chain of N coupled two-level systems. Panels (a, b, c): the results for a pair [cf. Eq. (S7)], a triple [cf. Eq. (S42)], and a quadruple of two-level systems respectively ($N = 2, 3, 4$). Dashed grey lines: bare transition frequencies.

while the associated eigenstates read [cf. Eq. (S8)]

$$||\psi_4\rangle\rangle = \frac{1}{\sqrt{\omega_1^2 + J^2}} \left(J|1,1\rangle - \omega_1|0,0\rangle \right), \tag{S13a}$$

$$||\psi_3\rangle\rangle = \frac{1}{\sqrt{2}} \left(|1,0\rangle + |0,1\rangle \right), \tag{S13b}$$

$$||\psi_2\rangle\rangle = \frac{1}{\sqrt{2}} \left(|1,0\rangle - |0,1\rangle \right), \tag{S13c}$$

$$||\psi_1\rangle\rangle = \frac{1}{\sqrt{\omega_4^2 + J^2}} \left(\omega_4|0,0\rangle - J|1,1\rangle \right). \tag{S13d}$$

Clearly, upon entering ultrastrong coupling the extremities of the ladder ω_1 and ω_4 become renormalized from their bare values, which arises due to the mixture of the bare states $|0,0\rangle$ and $|1,1\rangle$ inside $||\psi_1\rangle\rangle$ and $||\psi_4\rangle\rangle$. This is made possible by the non-number conserving processes described by the C-R terms. These energy ladder reconstructions are clearly visible in Fig. S2 (a), which displays the full eigenfrequencies ω_n as a function of J.

The dependence of the weightings of the bare states comprising the eigenstates $||\psi_n\rangle\rangle$ with the coupling strength J is represented in Fig. S3. In the figure white represents zero overlap, and increasingly dark red denotes increasingly large overlap. Noticeably, the intermediate states are unaffected by the magnitude of J, while the ground and highest energy states provide the fingerprints of ultrastrong coupling by breaking the conservation of the number of excitations.

C. Correlations

In terms of the Hamiltonian $\hat{H}$ given by Eq. (S1), the quantum Liouville-von Neumann equation for the density matrix ρ reads

$$\partial_t \rho = \mathrm{i} \left[\rho, \hat{H} \right]. \tag{S14}$$

Upon using the property $\langle \mathcal{O} \rangle = \mathrm{Tr}\,(\mathcal{O}\rho)$, which is valid for any operator $\mathcal{O}$, one may find the first and second moments of the system. We consider each case in turn.

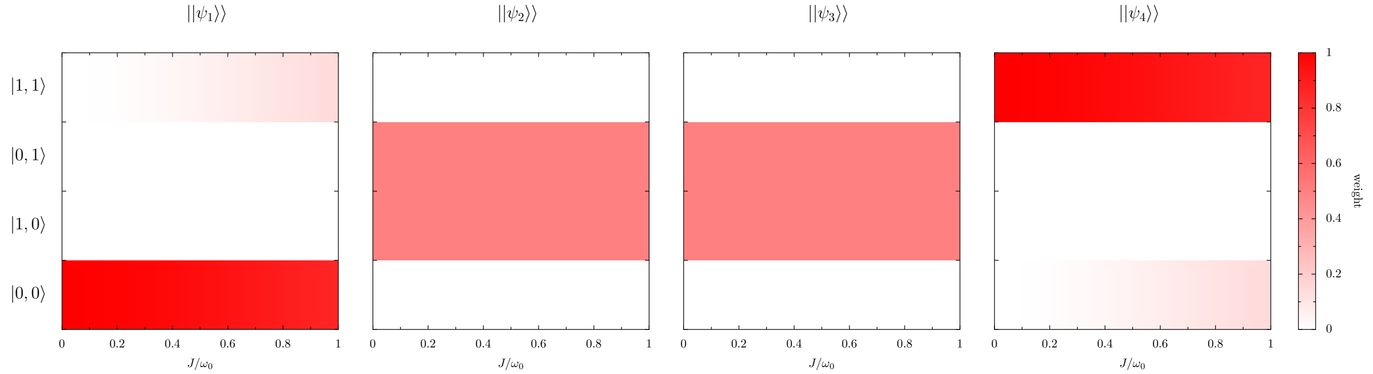

FIG. S3. **Fidelity of the pair eigenstates.** Weightings of the bare states, $|0,0\rangle$, $|1,0\rangle$, $|0,1\rangle$, and $|1,1\rangle$, which compose the dressed eigenstates $||\psi_n\rangle\rangle$ in the ultrastrong coupling regime [cf. Eq. (S13)], as a function of the coupling strength J (in units of the transition frequency ω_0). Color bar: weighting from 1 (red) to 0 (white).

First moments. The first moments of the system may be found from the equation of motion

$$\partial_t \mathbf{u} + \mathbf{M}\mathbf{u} = 0, \tag{S15}$$

where $\mathbf{u}$ collects the mean values of the operators, via

$$\mathbf{u} = \begin{pmatrix} \mathbf{v} \\ \mathbf{v}^\dagger \end{pmatrix}, \qquad \mathbf{v} = \begin{pmatrix} \langle \sigma_1 \rangle \\ \langle \sigma_2 \rangle \\ \langle \sigma_1^\dagger \sigma_1 \sigma_2 \rangle \\ \langle \sigma_1 \sigma_2^\dagger \sigma_2 \rangle \end{pmatrix}, \tag{S16}$$

and where the 8×8 dynamical matrix $\mathbf{M}$ is defined as

$$\mathbf{M} = \begin{pmatrix} \mathbf{M}_{11} & \mathbf{M}_{12} \\ \mathbf{M}_{12}^* & \mathbf{M}_{11}^* \end{pmatrix}, \tag{S17}$$

$$\mathbf{M}_{11} = \begin{pmatrix} \mathrm{i}\omega_0 & \mathrm{i}J & -2\mathrm{i}J & 0 \\ \mathrm{i}J & \mathrm{i}\omega_0 & 0 & -2\mathrm{i}J \\ 0 & 0 & \mathrm{i}\omega_0 & -\mathrm{i}J \\ 0 & 0 & -\mathrm{i}J & \mathrm{i}\omega_0 \end{pmatrix}, \qquad \mathbf{M}_{12} = \begin{pmatrix} 0 & \mathrm{i}J & -2\mathrm{i}J & 0 \\ \mathrm{i}J & 0 & 0 & -2\mathrm{i}J \\ \mathrm{i}J & 0 & 0 & -\mathrm{i}J \\ 0 & \mathrm{i}J & -\mathrm{i}J & 0 \end{pmatrix}. \tag{S18}$$

Subject to the initial condition of $\langle \sigma_1 \rangle = 1$ and $\langle \sigma_2 \rangle = 0$ at $t = 0$, the solution of Eq. (S15) yields the results

$$\langle \sigma_1 \rangle = \left\{ \cos\left(\tilde{\omega}_0 t\right) + \mathrm{i}\frac{\omega_0}{\tilde{\omega}_0} \sin\left(\tilde{\omega}_0 t\right) \right\} \cos\left(Jt\right), \tag{S19a}$$

$$\langle \sigma_2 \rangle = \mathrm{i}\left\{ \cos\left(\tilde{\omega}_0 t\right) + \mathrm{i}\frac{\omega_0}{\tilde{\omega}_0} \sin\left(\tilde{\omega}_0 t\right) \right\} \sin\left(Jt\right), \tag{S19b}$$

where the renormalized frequency $\tilde{\omega}_0$ is defined in Eq. (S12). The product of Eq. (S19) with their complex conjugate expressions for $\langle \sigma_1^\dagger \rangle$ and $\langle \sigma_2^\dagger \rangle$ yields Eq. (4) in the main text.

Second moments. Similarly, the second moments may be determined from the equation of motion

$$\partial_t \mathbf{w} = \mathbf{P} - \mathbf{Q}\mathbf{w}, \tag{S20}$$

where the mean values of the second moments $\mathbf{w}$, the effective drive term $\mathbf{P}$, and the 6×6 dynamical matrix $\mathbf{Q}$ are defined via

$$\mathbf{w} = \begin{pmatrix} \langle \sigma_1^\dagger \sigma_1 \rangle \\ \langle \sigma_2^\dagger \sigma_2 \rangle \\ \langle \sigma_1^\dagger \sigma_2 \rangle \\ \langle \sigma_2^\dagger \sigma_1 \rangle \\ \langle \sigma_1 \sigma_2 \rangle \\ \langle \sigma_1^\dagger \sigma_2^\dagger \rangle \end{pmatrix}, \qquad \mathbf{P} = \begin{pmatrix} 0 \\ 0 \\ 0 \\ 0 \\ -\mathrm{i}J \\ \mathrm{i}J \end{pmatrix}, \qquad \mathbf{Q} = \begin{pmatrix} 0 & 0 & \mathrm{i}J & -\mathrm{i}J & \mathrm{i}J & -\mathrm{i}J \\ 0 & 0 & -\mathrm{i}J & \mathrm{i}J & \mathrm{i}J & -\mathrm{i}J \\ \mathrm{i}J & -\mathrm{i}J & 0 & 0 & 0 & 0 \\ -\mathrm{i}J & \mathrm{i}J & 0 & 0 & 0 & 0 \\ -\mathrm{i}J & -\mathrm{i}J & 0 & 0 & 2\mathrm{i}\omega_0 & 0 \\ \mathrm{i}J & \mathrm{i}J & 0 & 0 & 0 & -2\mathrm{i}\omega_0 \end{pmatrix}. \tag{S21}$$

With the initial condition $\langle \sigma_1^\dagger \sigma_1 \rangle = 1$ and $\langle \sigma_2^\dagger \sigma_2 \rangle = 0$ at $t = 0$, the solution of Eq. (S20) yields

$$\langle \sigma_1^\dagger \sigma_1 \rangle = \cos^2(Jt), \qquad \langle \sigma_2^\dagger \sigma_2 \rangle = \sin^2(Jt), \qquad \langle \sigma_2^\dagger \sigma_1 \rangle = \langle \sigma_1^\dagger \sigma_2 \rangle^* = \frac{\mathrm{i}}{2}\sin(2Jt). \tag{S22}$$

The lack of equivalence between $\langle \sigma_n^\dagger \sigma_n \rangle$ and $\langle \sigma_n^\dagger \rangle \langle \sigma_n \rangle$ is due to corrections to the prior result of $\langle \sigma_n^\dagger \rangle \langle \sigma_n \rangle$.

D. Dynamics

The density matrix ρ of the two coupled qubits has $4^2 = 16$ elements, which may be appropriately written in matrix form

$$\rho = \begin{pmatrix} \rho_{0,0;\,0,0} & \rho_{0,0;\,1,0} & \rho_{0,0;\,0,1} & \rho_{0,0;\,1,1} \\ \rho_{1,0;\,0,0} & \rho_{1,0;\,1,0} & \rho_{1,0;\,0,1} & \rho_{1,0;\,1,1} \\ \rho_{0,1;\,0,0} & \rho_{0,1;\,1,0} & \rho_{0,1;\,0,1} & \rho_{0,1;\,1,1} \\ \rho_{1,1;\,0,0} & \rho_{1,1;\,1,0} & \rho_{1,1;\,0,1} & \rho_{1,1;\,1,1} \end{pmatrix}, \tag{S23}$$

where the matrix elements $\rho_{n,m;\,p,q} = \langle n,m|\rho|p,q \rangle$ span the basis of 4 pure states $|0,0\rangle$, $|1,0\rangle$, $|0,1\rangle$ and $|1,1\rangle$. The matrix elements $\rho_{n,m;\,p,q}$ may be found from Eq. (S14), leading to the following closed system of 8 equations for the principle elements of interest

$$\partial_t \begin{pmatrix} \rho_{0,0;\,0,0} \\ \rho_{1,0;\,1,0} \\ \rho_{0,1;\,0,1} \\ \rho_{1,1;\,1,1} \\ \rho_{1,0;\,0,1} \\ \rho_{0,1;\,1,0} \\ \rho_{0,0;\,1,1} \\ \rho_{1,1;\,0,0} \end{pmatrix} = \begin{pmatrix} 0 & 0 & 0 & 0 & 0 & 0 & \mathrm{i}J & -\mathrm{i}J \\ 0 & 0 & 0 & 0 & \mathrm{i}J & -\mathrm{i}J & 0 & 0 \\ 0 & 0 & 0 & 0 & -\mathrm{i}J & \mathrm{i}J & 0 & 0 \\ 0 & 0 & 0 & 0 & 0 & 0 & -\mathrm{i}J & \mathrm{i}J \\ 0 & \mathrm{i}J & -\mathrm{i}J & 0 & 0 & 0 & 0 & 0 \\ 0 & -\mathrm{i}J & \mathrm{i}J & 0 & 0 & 0 & 0 & 0 \\ \mathrm{i}J & 0 & 0 & -\mathrm{i}J & 0 & 0 & 2\mathrm{i}\omega_0 & 0 \\ -\mathrm{i}J & 0 & 0 & \mathrm{i}J & 0 & 0 & 0 & -2\mathrm{i}\omega_0 \end{pmatrix} \begin{pmatrix} \rho_{0,0;\,0,0} \\ \rho_{1,0;\,1,0} \\ \rho_{0,1;\,0,1} \\ \rho_{1,1;\,1,1} \\ \rho_{1,0;\,0,1} \\ \rho_{0,1;\,1,0} \\ \rho_{0,0;\,1,1} \\ \rho_{1,1;\,0,0} \end{pmatrix}, \tag{S24}$$

where the full Hamiltonian of Eq. (S1), complete with counter-rotating terms, was used. (If the RWA was instead employed, the equation analogous to Eq. (S24) would become 6-dimensional, where the final two rows and final two columns of the above matrix equation would have been discarded). Hence the density matrix ρ for the eight elements of interest, as well as its partial transpose $\rho^{\mathrm{T_P}}$, read

$$\rho = \begin{pmatrix} \rho_{0,0;\,0,0} & 0 & 0 & \rho_{0,0;\,1,1} \\ 0 & \rho_{1,0;\,1,0} & \rho_{1,0;\,0,1} & 0 \\ 0 & \rho_{0,1;\,1,0} & \rho_{0,1;\,0,1} & 0 \\ \rho_{1,1;\,0,0} & 0 & 0 & \rho_{1,1;\,1,1} \end{pmatrix}, \qquad \rho^{\mathrm{T_P}} = \begin{pmatrix} \rho_{0,0;\,0,0} & 0 & 0 & \rho_{0,1;\,1,0} \\ 0 & \rho_{1,0;\,1,0} & \rho_{1,1;\,0,1} & 0 \\ 0 & \rho_{0,0;\,1,1} & \rho_{0,1;\,0,1} & 0 \\ \rho_{1,0;\,0,1} & 0 & 0 & \rho_{1,1;\,1,1} \end{pmatrix}. \tag{S25}$$

The latter quantity $\rho^{\mathrm{T_P}}$ appears indirectly, via its eigenvalues λ_n, in the definition of the negativity $\mathcal{N}$

$$\mathcal{N} = \sum_n \frac{|\lambda_n| - \lambda_n}{2}, \tag{S26}$$

which is a measure of quantum entanglement.

Single-excitation sector. The Hamiltonian of Eq. (S1) ensures that the single-excitation sector is unlinked from all other excitation sectors, such that it can be removed from Eq. (S14). This procedure leads to the following 4×4 matrix equation, wholly dependent on J,

$$\partial_t \begin{pmatrix} \rho_{1,0;\,1,0} \\ \rho_{0,1;\,0,1} \\ \rho_{1,0;\,0,1} \\ \rho_{0,1;\,1,0} \end{pmatrix} = \begin{pmatrix} 0 & 0 & \mathrm{i}J & -\mathrm{i}J \\ 0 & 0 & -\mathrm{i}J & \mathrm{i}J \\ \mathrm{i}J & -\mathrm{i}J & 0 & 0 \\ -\mathrm{i}J & \mathrm{i}J & 0 & 0 \end{pmatrix} \begin{pmatrix} \rho_{1,0;\,1,0} \\ \rho_{0,1;\,0,1} \\ \rho_{1,0;\,0,1} \\ \rho_{0,1;\,1,0} \end{pmatrix}. \tag{S27}$$

With the initial condition of a populated $|1,0\rangle$ state only ($\rho_{1,0;\,1,0} = 1$, $\rho_{0,1;\,0,1} = \rho_{1,0;\,0,1} = \rho_{0,1;\,1,0} = 0$ at time $t = 0$), Eq. (S27) yields the solutions

$$\rho_{1,0;\,1,0} = \cos^2(Jt), \qquad \rho_{0,1;\,0,1} = \sin^2(Jt), \qquad \rho_{1,0;\,0,1} = \frac{\mathrm{i}}{2}\sin(2Jt), \qquad \rho_{0,1;\,1,0} = -\frac{\mathrm{i}}{2}\sin(2Jt), \tag{S28}$$

which are the same solutions with and without employing the RWA in the Hamiltonian $\hat{H}$ appearing in Eq. (S14). These standard trigonometric results describe the population dynamics across the $|1,0\rangle$ and $|0,1\rangle$ states, as well as the complex coherences. The negativity $\mathcal{N}$, measuring the degree of entanglement of the system, follows from Eq. (S26) as

$$\mathcal{N} = \frac{1}{2}\Big| \sin\left(2Jt\right) \Big|, \tag{S29}$$

describing sinusoidal oscillations of amplitude $1/2$.

Zero- and two-excitation sectors. The counter-rotating terms in the Hamiltonian of Eq. (S1) link the zero-excitation and two-excitation sectors, so that these two sectors may be removed from Eq. (S24) together. This regrouping procedure yields the following 4-dimensional matrix equation, dependent on both J and ω_0 [cf. Eq. (S27)]

$$\partial_t \begin{pmatrix} \rho_{0,0;\,0,0} \\ \rho_{1,1;\,1,1} \\ \rho_{0,0;\,1,1} \\ \rho_{1,1;\,0,0} \end{pmatrix} = \begin{pmatrix} 0 & 0 & \mathrm{i}J & -\mathrm{i}J \\ 0 & 0 & -\mathrm{i}J & \mathrm{i}J \\ \mathrm{i}J & -\mathrm{i}J & 2\mathrm{i}\omega_0 & 0 \\ -\mathrm{i}J & \mathrm{i}J & 0 & -2\mathrm{i}\omega_0 \end{pmatrix} \begin{pmatrix} \rho_{0,0;\,0,0} \\ \rho_{1,1;\,1,1} \\ \rho_{0,0;\,1,1} \\ \rho_{1,1;\,0,0} \end{pmatrix}. \tag{S30}$$

Choosing the initial condition of the zero-excitation state ($\rho_{0,0;\,0,0} = 1$, $\rho_{1,1;\,1,1} = \rho_{0,0;\,1,1} = \rho_{1,1;\,0,0} = 0$ at time $t = 0$), Eq. (S30) leads to the solutions

$$\rho_{0,0;\,0,0} = \frac{\omega_0^2}{\tilde{\omega}_0^2} + \frac{J^2}{\tilde{\omega}_0^2}\cos^2\left(\tilde{\omega}_0 t\right), \qquad \rho_{1,1;\,1,1} = \frac{J^2}{\tilde{\omega}_0^2}\sin^2\left(\tilde{\omega}_0 t\right), \tag{S31}$$

$$\rho_{0,0;\,1,1} = \frac{1}{2}\frac{J}{\tilde{\omega}_0}\left\{ \frac{\omega_0}{\tilde{\omega}_0}\cos\left(2\tilde{\omega}_0 t\right) - \frac{\omega_0}{\tilde{\omega}_0} + \mathrm{i}\sin\left(2\tilde{\omega}_0 t\right) \right\}, \qquad \rho_{1,1;\,0,0} = \frac{1}{2}\frac{J}{\tilde{\omega}_0}\left\{ \frac{\omega_0}{\tilde{\omega}_0}\cos\left(2\tilde{\omega}_0 t\right) - \frac{\omega_0}{\tilde{\omega}_0} - \mathrm{i}\sin\left(2\tilde{\omega}_0 t\right) \right\}, \tag{S32}$$

where $\tilde{\omega}_0$ is defined in Eq. (S12). The results of Eq. (S31) highlight how the C-R terms lead to the $|1,1\rangle$ state becoming partially occupied, with the fraction $J^2/(\omega_0^2 + J^2)$ determining the amplitude of the sinusoidal oscillations, in stark contrast to what happens in the RWA. The degree of entanglement of the system can be computed using the negativity $\mathcal{N}$, as defined in Eq. (S26), leading to the markedly nonzero expression

$$\mathcal{N} = \frac{J}{\tilde{\omega}_0}\sqrt{\left(\frac{\omega_0^2 + J^2\cos^2\left(\tilde{\omega}_0 t\right)}{\tilde{\omega}_0^2}\right)\sin^2\left(\tilde{\omega}_0 t\right)}, \tag{S33}$$

which arises solely due to the C-R terms (in the RWA, one would instead have $\mathcal{N} = 0$ here). In the limit of $J \ll \omega_0$, Eq. (S34) reduces to the much simpler form

$$\mathcal{N} \simeq \frac{J}{\omega_0}\Big| \sin\left(\omega_0 t\right) \Big|, \qquad J \ll \omega_0, \tag{S34}$$

showcasing the J/ω_0 amplitude of the negativity, and the fast sinusoidal oscillations in the dimensionless variable $\omega_0 t$.

II. A TRIPLE OF COUPLED TWO-LEVEL SYSTEMS

To build up further intuition, let us now consider a triple of coupled 2LSs, as captured by the Hamiltonian

$$\hat{H} = \omega_0\left(\sigma_1^\dagger\sigma_1 + \sigma_2^\dagger\sigma_2 + \sigma_3^\dagger\sigma_3\right) + J\left(\sigma_1^\dagger\sigma_2 + \sigma_2^\dagger\sigma_1 + \sigma_1\sigma_2 + \sigma_1^\dagger\sigma_2^\dagger + \sigma_2^\dagger\sigma_3 + \sigma_3^\dagger\sigma_2 + \sigma_2\sigma_3 + \sigma_2^\dagger\sigma_3^\dagger\right). \tag{S35}$$

Where $\mathcal{N}$ counts the number of excitations, the bare state basis may be defined via

$$\mathcal{N} = 0 \qquad |0,0,0\rangle, \tag{S36a}$$
$$\mathcal{N} = 1 \qquad \sigma_1^\dagger|0,0,0\rangle = |1,0,0\rangle, \qquad \sigma_2^\dagger|0,0,0\rangle = |0,1,0\rangle, \qquad \sigma_3^\dagger|0,0,0\rangle = |0,0,1\rangle, \tag{S36b}$$
$$\mathcal{N} = 2 \qquad \sigma_1^\dagger\sigma_2^\dagger|0,0,0\rangle = |1,1,0\rangle, \qquad \sigma_1^\dagger\sigma_3^\dagger|0,0,0\rangle = |1,0,1\rangle, \qquad \sigma_2^\dagger\sigma_3^\dagger|0,0,0\rangle = |0,1,1\rangle, \tag{S36c}$$
$$\mathcal{N} = 3 \qquad \sigma_1^\dagger\sigma_2^\dagger\sigma_3^\dagger|0,0,0\rangle = |1,1,1\rangle, \tag{S36d}$$

such that the 8×8 matrix representation of Eq. (S36) readily follows as

$$H = \begin{pmatrix} 0 & 0 & 0 & 0 & J & 0 & J & 0 \\ 0 & \omega_0 & J & 0 & 0 & 0 & 0 & J \\ 0 & J & \omega_0 & J & 0 & 0 & 0 & 0 \\ 0 & 0 & J & \omega_0 & 0 & 0 & 0 & J \\ J & 0 & 0 & 0 & 2\omega_0 & J & 0 & 0 \\ 0 & 0 & 0 & 0 & J & 2\omega_0 & J & 0 \\ J & 0 & 0 & 0 & 0 & J & 2\omega_0 & 0 \\ 0 & J & 0 & J & 0 & 0 & 0 & 3\omega_0 \end{pmatrix}. \tag{S37}$$

A. Strong coupling

Within the RWA, the number of excitations is conserved so that Eq. (S37) reduces to the block diagonal form

$$H' = \mathrm{diag}\{H'_0, H'_1, H'_2, H'_3\}, \tag{S38}$$

which is composed of the submatrices describing the 0, 1, 2, and 3 excitation sectors, where

$$H'_0 = 0, \qquad H'_1 = \begin{pmatrix} \omega_0 & J & 0 \\ J & \omega_0 & J \\ 0 & J & \omega_0 \end{pmatrix}, \qquad H'_2 = \begin{pmatrix} 2\omega_0 & J & 0 \\ J & 2\omega_0 & J \\ 0 & J & 2\omega_0 \end{pmatrix}, \qquad H'_3 = 3\omega_0. \tag{S39}$$

The eigenfrequencies ω'_n are as follows

$$\omega'_8 = 3\omega_0, \tag{S40a}$$
$$\omega'_7 = 2\omega_0 + \sqrt{2}J, \tag{S40b}$$
$$\omega'_6 = 2\omega_0, \tag{S40c}$$
$$\omega'_5 = 2\omega_0 - \sqrt{2}J, \tag{S40d}$$
$$\omega'_4 = \omega_0 + \sqrt{2}J, \tag{S40e}$$
$$\omega'_3 = \omega_0, \tag{S40f}$$
$$\omega'_2 = \omega_0 - \sqrt{2}J, \tag{S40g}$$
$$\omega'_1 = 0, \tag{S40h}$$

and are plotted in Fig. S1 (b), where the differences to immediately smaller and immediately larger chains may be seen in the panels either side of panel (b).

B. Ultrastrong coupling

The full governing matrix of Eq. (S37) can be reshuffled into two matrices, one describing the connected zero and two excitation subspace, and the other describing the one and three excitation subspace, as follows

$$H_{0\leftrightarrow 2} = \begin{pmatrix} 0 & J & 0 & J \\ J & 2\omega_0 & J & 0 \\ 0 & J & 2\omega_0 & J \\ J & 0 & J & 2\omega_0 \end{pmatrix}, \qquad H_{1\leftrightarrow 3} = \begin{pmatrix} \omega_0 & J & 0 & J \\ J & \omega_0 & J & 0 \\ 0 & J & \omega_0 & J \\ J & 0 & J & 3\omega_0 \end{pmatrix}, \tag{S41}$$

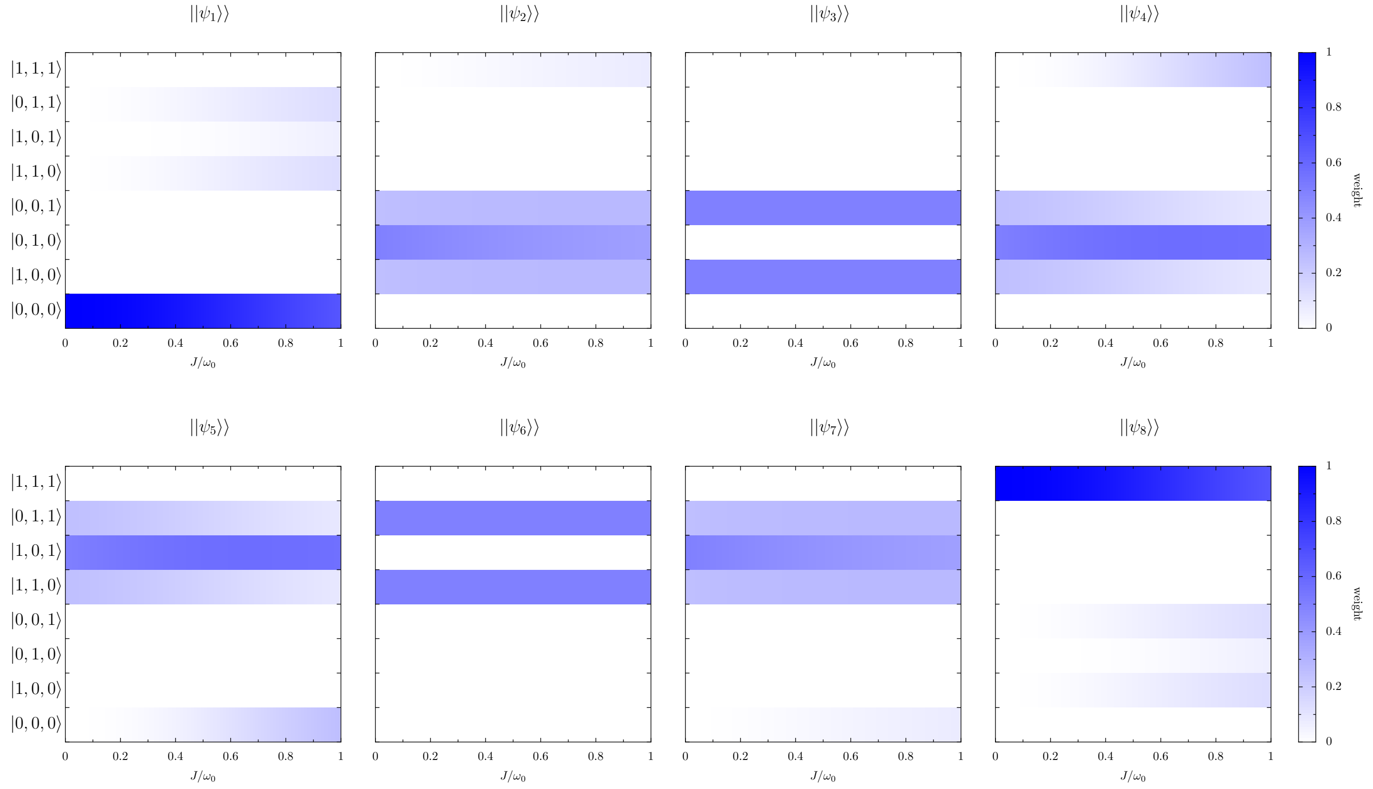

FIG. S4. **Fidelity of the triple eigenstates.** Weightings of the bare states in the dressed eigenstates $||\psi_n\rangle\rangle$ in the ultrastrong coupling regime, as a function of the coupling strength J (in units of the transition frequency ω_0). Color bar: weighting from 1 (blue) to 0 (white).

which yield exact expressions for the eigenfrequencies ω_n

$$\omega_8 = \frac{1}{3}\left[5\omega_0 + 4\sqrt{\omega_0^2 + 3J^2}\cos\left(\frac{\phi}{3}\right)\right], \tag{S42a}$$

$$\omega_7 = \frac{4}{3}\left[\omega_0 + \sqrt{\omega_0^2 + 3J^2}\cos\left(\frac{\phi}{3}\right)\right], \tag{S42b}$$

$$\omega_6 = 2\omega_0, \tag{S42c}$$

$$\omega_5 = \frac{4}{3}\left[\omega_0 + \sqrt{\omega_0^2 + 3J^2}\cos\left(\frac{\phi + 2\pi}{3}\right)\right], \tag{S42d}$$

$$\omega_4 = \frac{1}{3}\left[5\omega_0 + 4\sqrt{\omega_0^2 + 3J^2}\cos\left(\frac{\phi + 4\pi}{3}\right)\right], \tag{S42e}$$

$$\omega_3 = \omega_0, \tag{S42f}$$

$$\omega_2 = \frac{1}{3}\left[5\omega_0 + 4\sqrt{\omega_0^2 + 3J^2}\cos\left(\frac{\phi + 2\pi}{3}\right)\right], \tag{S42g}$$

$$\omega_1 = \frac{4}{3}\left[\omega_0 + \sqrt{\omega_0^2 + 3J^2}\cos\left(\frac{\phi + 4\pi}{3}\right)\right], \tag{S42h}$$

where we have introduced the angular parameter

$$\phi = \arccos\left(\frac{\omega_0}{4}\frac{4\omega_0^2 - 9J^2}{[\omega_0^2 + 3J^2]^{\frac{3}{2}}}\right). \tag{S43}$$

These eigenfrequencies ω_n are plotted in Fig. S2 (b) as a function of J, with complementary results available in the panels either side for shorter and longer chains. The fidelities of the eigenstates $||\psi_n\rangle\rangle$ as a function of J are shown in Fig. S4, with the ordering from the ground state $||\psi_1\rangle\rangle$ to the fully-occupied state $||\psi_8\rangle\rangle$. The influence of ultrastrong coupling is demonstrated in the breakdown of the excitation number conserving sectors.

III. A QUADRUPLE OF COUPLED TWO-LEVEL SYSTEMS

The final short chain that we shall consider is the quadruple of coupled 2LSs, which has the Hamiltonian

$$\hat{H} = \omega_0 \left(\sigma_1^\dagger\sigma_1 + \sigma_2^\dagger\sigma_2 + \sigma_3^\dagger\sigma_3 + \sigma_4^\dagger\sigma_4\right) \\ + J\left(\sigma_1^\dagger\sigma_2 + \sigma_2^\dagger\sigma_1 + \sigma_1\sigma_2 + \sigma_1^\dagger\sigma_2^\dagger + \sigma_2^\dagger\sigma_3 + \sigma_3^\dagger\sigma_2 + \sigma_2\sigma_3 + \sigma_2^\dagger\sigma_3^\dagger + \sigma_3^\dagger\sigma_4 + \sigma_4^\dagger\sigma_3 + \sigma_3\sigma_4 + \sigma_3^\dagger\sigma_4^\dagger\right), \tag{S44}$$

and the 16-dimensional bare state basis, ordered by the excitation number, readily follows as

$$|\mathbf{0}\rangle = |0,0,0,0\rangle, \tag{S45a}$$
$$\sigma_1^\dagger|\mathbf{0}\rangle = |1,0,0,0\rangle,\ \sigma_2^\dagger|\mathbf{0}\rangle = |0,1,0,0\rangle,\ \sigma_3^\dagger|\mathbf{0}\rangle = |0,0,1,0\rangle,\ \sigma_4^\dagger|\mathbf{0}\rangle = |0,0,0,1\rangle, \tag{S45b}$$
$$\sigma_1^\dagger\sigma_2^\dagger|\mathbf{0}\rangle = |1,1,0,0\rangle,\ \sigma_1^\dagger\sigma_3^\dagger|\mathbf{0}\rangle = |1,0,1,0\rangle, \sigma_1^\dagger\sigma_4^\dagger|\mathbf{0}\rangle = |1,0,0,1\rangle, \\ \sigma_2^\dagger\sigma_3^\dagger|\mathbf{0}\rangle = |0,1,1,0\rangle,\ \sigma_2^\dagger\sigma_4^\dagger|\mathbf{0}\rangle = |0,1,0,1\rangle,\ \sigma_3^\dagger\sigma_4^\dagger|\mathbf{0}\rangle = |0,0,1,1\rangle, \tag{S45c}$$
$$\sigma_1^\dagger\sigma_2^\dagger\sigma_3^\dagger|\mathbf{0}\rangle = |1,1,1,0\rangle,\ \sigma_1^\dagger\sigma_2^\dagger\sigma_4^\dagger|\mathbf{0}\rangle = |1,1,0,1\rangle,\ \sigma_1^\dagger\sigma_3^\dagger\sigma_4^\dagger|\mathbf{0}\rangle = |1,0,1,1\rangle,\ \sigma_2^\dagger\sigma_3^\dagger\sigma_4^\dagger|\mathbf{0}\rangle = |0,1,1,1\rangle, \tag{S45d}$$
$$\sigma_1^\dagger\sigma_2^\dagger\sigma_3^\dagger\sigma_4^\dagger|\mathbf{0}\rangle = |1,1,1,1\rangle. \tag{S45e}$$

A. Strong coupling

In the RWA, the matrix representation of Eq. (S44) can be neatly written as the block diagonal Hamiltonian

$$H' = \text{diag}\{H_0', H_1', H_2', H_3', H_4'\}, \tag{S46}$$

where we define the sub-matrices H_n' as follows

$$H_0' = 0, \qquad H_1' = \begin{pmatrix} \omega_0 & J & 0 & 0 \\ J & \omega_0 & J & 0 \\ 0 & J & \omega_0 & J \\ 0 & 0 & J & \omega_0 \end{pmatrix}, \qquad H_2' = \begin{pmatrix} 2\omega_0 & J & 0 & 0 & 0 & 0 \\ J & 2\omega_0 & J & J & 0 & 0 \\ 0 & J & 2\omega_0 & 0 & J & 0 \\ 0 & J & 0 & 2\omega_0 & J & 0 \\ 0 & 0 & J & J & 2\omega_0 & J \\ 0 & 0 & 0 & 0 & J & 2\omega_0 \end{pmatrix}, \tag{S47}$$

$$H_3' = \begin{pmatrix} 3\omega_0 & J & 0 & 0 \\ J & 3\omega_0 & J & 0 \\ 0 & J & 3\omega_0 & J \\ 0 & 0 & J & 3\omega_0 \end{pmatrix}, \qquad H_4' = 4\omega_0. \tag{S48}$$

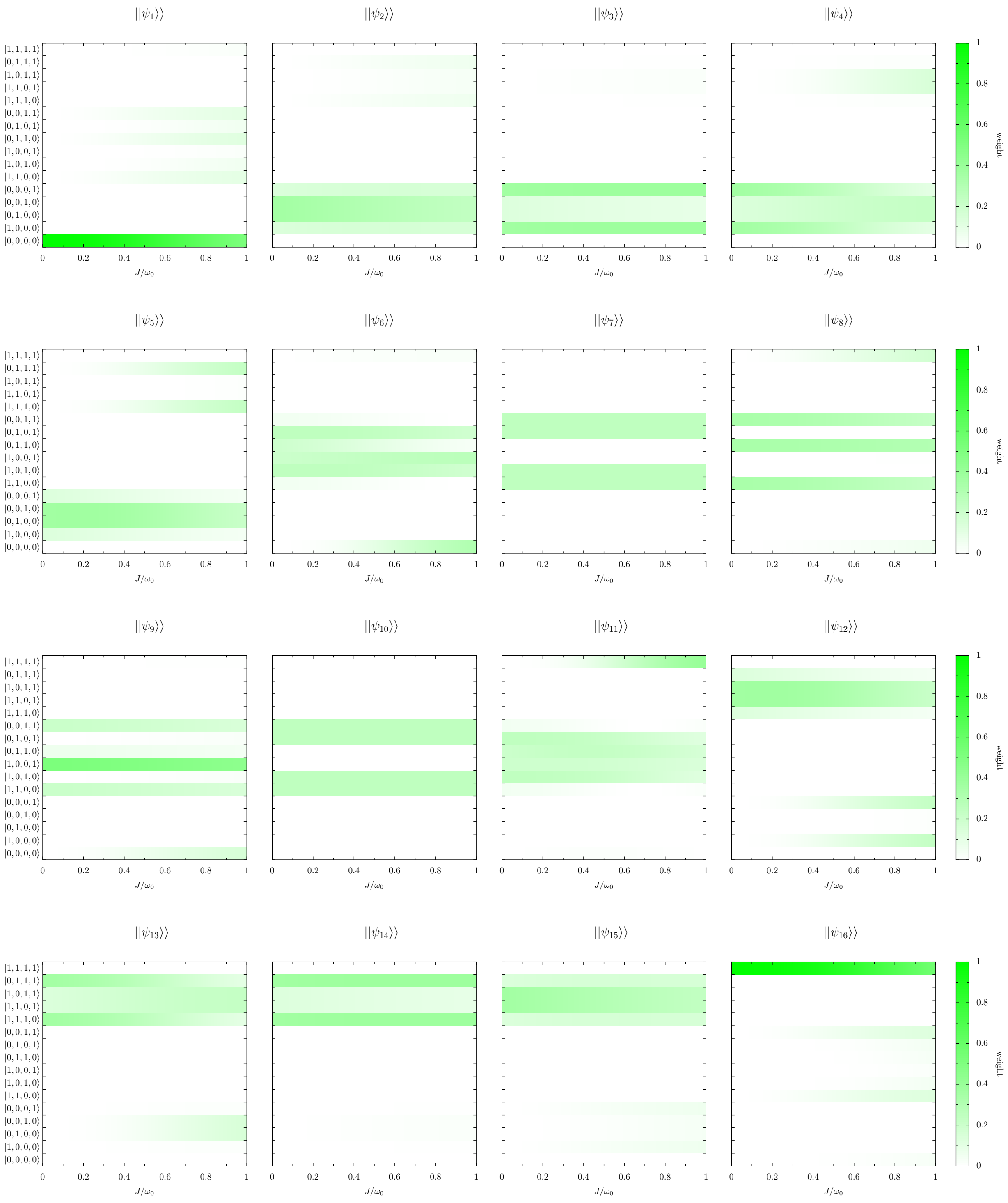

FIG. S5. **Quadruple eigenstates.** Weightings of the bare states in the dressed eigenstates $||\psi_n\rangle\rangle$ in the ultrastrong coupling regime, as a function of the coupling strength J (in units of the transition frequency ω_0). Color bar: weighting from 1 (green) to 0 (white).

The strong coupling eigenfrequencies ω'_n follow directly as

$$\omega'_{16} = 4\omega_0, \tag{S49a}$$
$$\omega'_{15} = 3\omega_0 + \left(\frac{\sqrt{5}+1}{2}\right) J, \tag{S49b}$$
$$\omega'_{14} = 3\omega_0 + \left(\frac{\sqrt{5}-1}{2}\right) J, \tag{S49c}$$
$$\omega'_{13} = 3\omega_0 - \left(\frac{\sqrt{5}-1}{2}\right) J, \tag{S49d}$$
$$\omega'_{12} = 3\omega_0 - \left(\frac{\sqrt{5}+1}{2}\right) J, \tag{S49e}$$
$$\omega'_{11} = 2\omega_0 + \sqrt{5}J, \tag{S49f}$$
$$\omega'_{10} = 2\omega_0 + J, \tag{S49g}$$
$$\omega'_{9} = 2\omega_0, \tag{S49h}$$
$$\omega'_{8} = 2\omega_0, \tag{S49i}$$
$$\omega'_{7} = 2\omega_0 - J, \tag{S49j}$$
$$\omega'_{6} = 2\omega_0 - \sqrt{5}J, \tag{S49k}$$
$$\omega'_{5} = \omega_0 + \left(\frac{\sqrt{5}+1}{2}\right) J, \tag{S49l}$$
$$\omega'_{4} = \omega_0 + \left(\frac{\sqrt{5}-1}{2}\right) J, \tag{S49m}$$
$$\omega'_{3} = \omega_0 - \left(\frac{\sqrt{5}-1}{2}\right) J, \tag{S49n}$$
$$\omega'_{2} = \omega_0 - \left(\frac{\sqrt{5}+1}{2}\right) J, \tag{S49o}$$
$$\omega'_{1} = 0, \tag{S49p}$$

and are plotted in Fig. S1 (c), alongside the results for smaller chains in panels (a) and (b).

B. Ultrastrong coupling

The C-R terms in the full Hamiltonian of Eq. (S44) impose that the odd-numbered and even-numbered excitation sectors must be neatly split into two, as follows

$$H_{0\leftrightarrow 2\leftrightarrow 4} = \begin{pmatrix} 0 & J & 0 & 0 & J & 0 & J & 0 \\ J & 2\omega_0 & J & 0 & 0 & 0 & 0 & J \\ 0 & J & 2\omega_0 & J & J & 0 & 0 & 0 \\ 0 & 0 & J & 2\omega_0 & 0 & J & 0 & J \\ J & 0 & J & 0 & 2\omega_0 & J & 0 & 0 \\ 0 & 0 & 0 & J & J & 2\omega_0 & J & 0 \\ J & 0 & 0 & 0 & 0 & J & 2\omega_0 & J \\ 0 & J & 0 & J & 0 & 0 & J & 4\omega_0 \end{pmatrix}, \qquad H_{1\leftrightarrow 3} = \begin{pmatrix} \omega_0 & J & 0 & 0 & J & 0 & J & 0 \\ J & \omega_0 & J & 0 & 0 & 0 & 0 & J \\ 0 & J & \omega_0 & J & J & 0 & 0 & 0 \\ 0 & 0 & J & \omega_0 & 0 & J & 0 & J \\ J & 0 & J & 0 & 3\omega_0 & J & 0 & 0 \\ 0 & 0 & 0 & J & J & 3\omega_0 & J & 0 \\ J & 0 & 0 & 0 & 0 & J & 3\omega_0 & J \\ 0 & J & 0 & J & 0 & 0 & J & 3\omega_0 \end{pmatrix}. \tag{S50}$$

The resulting eigenvalues are plotted in Fig. S2 (c), where information about smaller chains is presented in panels (a) and (b). The make-up of the eigenstates $||\psi_n\rangle\rangle$ in terms of the bare states is shown in Fig. S5 as a function of J.

IV. A CHAIN OF COUPLED TWO-LEVEL SYSTEMS

Let us now consider the more interesting case of a general linear chain, that of N coupled 2LSs. The Hamiltonian $\hat{H}$ reads

$$\hat{H} = \omega_0 \sum_{n=1}^{N} \sigma_n^\dagger \sigma_n + J \sum_{n=1}^{N-1} \left(\sigma_n + \sigma_n^\dagger\right)\left(\sigma_{n+1} + \sigma_{n+1}^\dagger\right), \tag{S51}$$

and the exponentially growing Hilbert space ensures an unwieldly $2^N \times 2^N$ matrix representation.

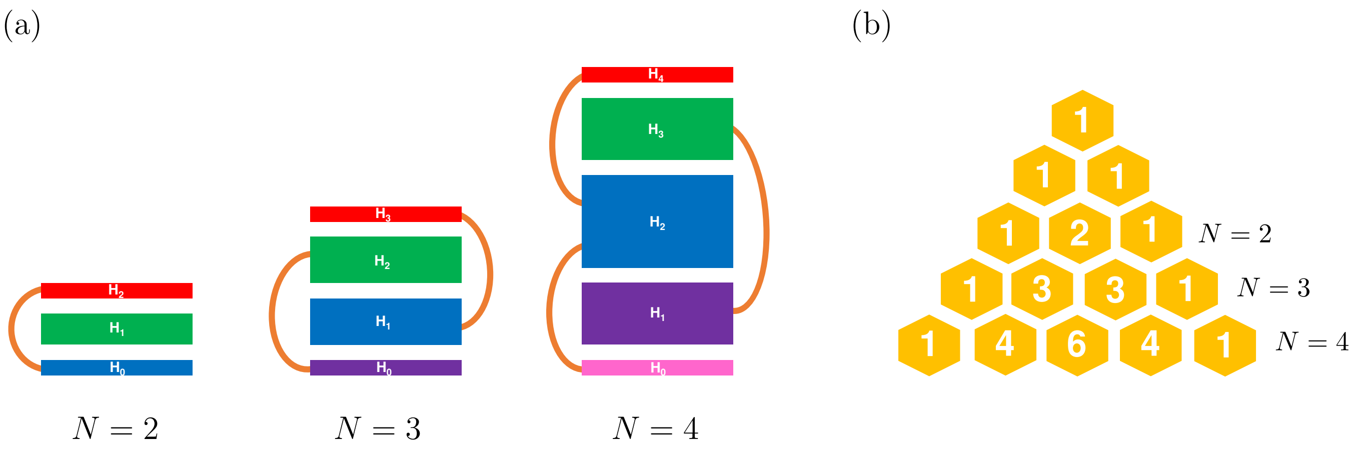

FIG. S6. **Structure of the Hamiltonian of a chain of N coupled two-level systems.** Panel (a): in the strong coupling regime, where the RWA is applied, the Hamiltonian may be split into constituent Hamiltonians $H_{\mathcal{N}}$, describing each $\mathcal{N}$-excitation subspace. In the ultrastrong coupling regime, where the RWA is not applied, the even and odd constituent Hamiltonians $H_{\mathcal{N}}$ respectively become linked (orange lines). Panel (b): the Hilbert space of the Hamiltonian is of size 2^N, while the size of the constituent Hamiltonian $H_{\mathcal{N}}$ in the RWA follows Pascal's triangle, being the binomial coefficient $N!/\mathcal{N}!/(N-\mathcal{N})!$.

A. Strong coupling

In the RWA, some general properties can nevertheless be drawn. Since $\hat{H}$ becomes number conserving after discarding the non-resonant terms, the resulting Hamiltonian may be split into chunks describing a different number of excitations $\mathcal{N}$, as is drawn in Fig. S6 (a) for small chains of size N. The size of each chunk is given by the binomial coefficient $N!/\mathcal{N}!/(N-\mathcal{N})!$, such that the structure follows Pascal's triangle, as is sketched in Fig. S6 (b).

Now let us consider the energy ladder of a general chain of size N. The $\mathcal{N}=0$ excitation sector remains trivial, with an empty ground state at zero energy, as one would expect. The $\mathcal{N}=1$ excitation sector corresponds to the standard cosine result of a one-dimensional tight-binding array. At the other end of the energy ladder, the $\mathcal{N}=N$ excitation sector is associated with the highest possible eigenfrequency $N\omega_0$, corresponding to a wholly excited, saturated state. The penultimate rung of the ladder, the $\mathcal{N}=N-1$ excitation sector, is a mirror image of the $\mathcal{N}=1$ case, with holes (or the absence of excitations) playing the role of excitations [it is this mirroring quality which allows for the so-called anti-edge states for a dimerized chain to emerge, as discussed in the main text]. These aforementioned results can be briefly listed as

$$\mathcal{N}=N, \qquad \omega'_{2^N}=N\omega_0, \tag{S52a}$$

$$\mathcal{N}=N-1, \qquad \omega'_{\ell}=(N-1)\,\omega_0+2J\cos\left(\pi\frac{[\ell-2^N+N+1]}{N+1}\right), \quad \ell=2^N-N,\dots,2^N-1. \tag{S52b}$$

$$\vdots$$

$$\mathcal{N}=1, \qquad \omega'_{m}=\omega_0+2J\cos\left(\pi\frac{[m-1]}{N+1}\right), \quad m=2,\dots,N+1. \tag{S52c}$$

$$\mathcal{N}=0, \qquad \omega'_{1}=0. \tag{S52d}$$

Elsewhere, in the intermediate rungs of the energy ladder, general analytic results are harder to write down.

B. Ultrastrong coupling

The C-R terms break the number conservation enjoyed in strong coupling. As shown in Fig. S6 (a), now all even-numbered and all odd-numbered excitation sectors are linked. In Fig. S7 we compare the eigenfrequencies in the strong (thick yellow lines) and ultrastrong (thin red lines) coupling regimes, as a function of the coupling strength J, for shorter chains from $N=2$ to $N=5$.

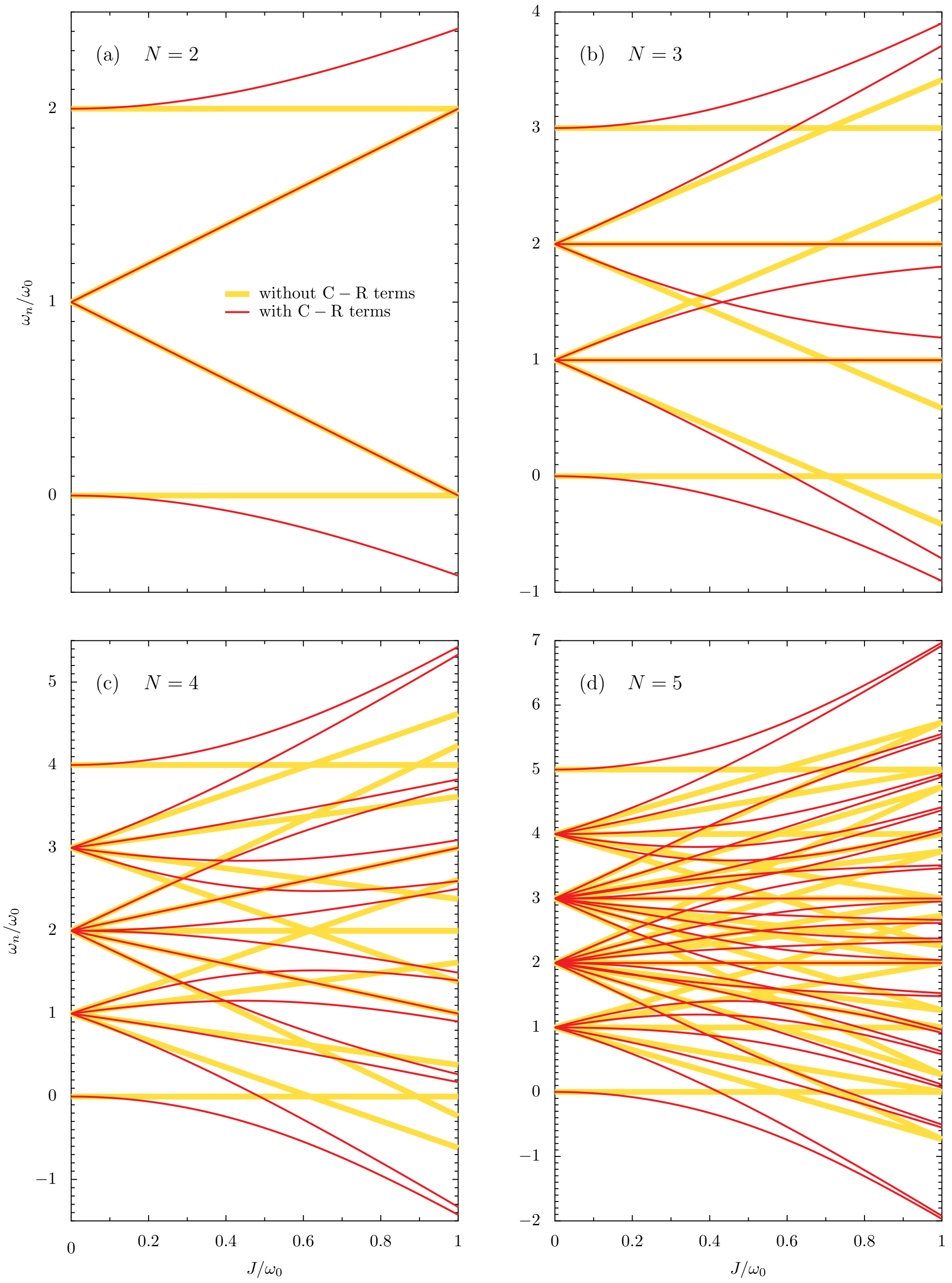

FIG. S7. **Eigenfrequencies of a chain of coupled two-level systems.** Eigenfrequencies in the strong (thick yellow lines) and ultrastrong (thin red lines) coupling regimes, as a function of the coupling strength J (in units of the transition frequency ω_0). We consider a chain of N coupled two-level systems with (a) $N = 2$, (b) $N = 3$, (c) $N = 4$, and (d) $N = 5$.

V. A DIMERIZED TRIPLE OF COUPLED TWO-LEVEL SYSTEMS

The main text considers a general dimerized chain. Here we complement those results by looking at the shortest possible dimerized chain, that of a triple of coupled two-level systems. The Hamiltonian $\hat{H}$ reads

$$\hat{H} = \omega_0 \left(\sigma_1^\dagger \sigma_1 + \sigma_2^\dagger \sigma_2 + \sigma_3^\dagger \sigma_3 \right) + J_1 \left(\sigma_1^\dagger \sigma_2 + \sigma_2^\dagger \sigma_1 + \sigma_1 \sigma_2 + \sigma_1^\dagger \sigma_2^\dagger \right) + J_2 \left(\sigma_2^\dagger \sigma_3 + \sigma_3^\dagger \sigma_2 + \sigma_2 \sigma_3 + \sigma_2^\dagger \sigma_3^\dagger \right), \tag{S53}$$

and the 8×8 matrix representation follows as [cf. Eq. (S37)]

$$H = \begin{pmatrix} 0 & 0 & 0 & 0 & J_1 & 0 & J_2 & 0 \\ 0 & \omega_0 & J_1 & 0 & 0 & 0 & 0 & J_2 \\ 0 & J_1 & \omega_0 & J_2 & 0 & 0 & 0 & 0 \\ 0 & 0 & J_2 & \omega_0 & 0 & 0 & 0 & J_1 \\ J_1 & 0 & 0 & 0 & 2\omega_0 & J_2 & 0 & 0 \\ 0 & 0 & 0 & 0 & J_2 & 2\omega_0 & J_1 & 0 \\ J_2 & 0 & 0 & 0 & 0 & J_1 & 2\omega_0 & 0 \\ 0 & J_2 & 0 & J_1 & 0 & 0 & 0 & 3\omega_0 \end{pmatrix}. \tag{S54}$$

A. Strong coupling

The RWA allows for Eq. (S54) to be simplified into the block diagonal form due to its number-conserving nature

$$H' = \text{diag}\{H'_0, H'_1, H'_2, H'_3\}, \tag{S55}$$

where the constituent Hamiltonians H'_n read

$$H'_0 = 0, \qquad H'_1 = \begin{pmatrix} \omega_0 & J_1 & 0 \\ J_1 & \omega_0 & J_2 \\ 0 & J_2 & \omega_0 \end{pmatrix}, \qquad H'_2 = \begin{pmatrix} 2\omega_0 & J_2 & 0 \\ J_2 & 2\omega_0 & J_1 \\ 0 & J_1 & 2\omega_0 \end{pmatrix}, \qquad H'_3 = 3\omega_0, \tag{S56}$$

leading to the set of RWA eigenfrequencies ω'_n [cf. Eq. (S40)]

$$\omega'_8 = 3\omega_0, \tag{S57a}$$

$$\omega'_7 = 2\omega_0 + \sqrt{J_1^2 + J_2^2}, \tag{S57b}$$

$$\omega'_6 = 2\omega_0, \tag{S57c}$$

$$\omega'_5 = 2\omega_0 - \sqrt{J_1^2 + J_2^2}, \tag{S57d}$$

$$\omega'_4 = \omega_0 + \sqrt{J_1^2 + J_2^2}, \tag{S57e}$$

$$\omega'_3 = \omega_0, \tag{S57f}$$

$$\omega'_2 = \omega_0 - \sqrt{J_1^2 + J_2^2}, \tag{S57g}$$

$$\omega'_1 = 0. \tag{S57h}$$

The RWA eigenfrequencies ω'_n are plotted in Fig. S8 as thick yellow lines, as a function of the dimerization parameter ϵ, where $\epsilon = (J_1 - J_2)/\bar{J}$. Each panel shows a different coupling strength $\bar{J}$, where $\bar{J} = J_1 + J_2$.

B. Ultrastrong coupling

Without taking the RWA, the ultrastrong coupling regime is governed by an even number of excitations Hamiltonian $H_{0\leftrightarrow 2}$, and its odd parter Hamiltonian $H_{1\leftrightarrow 3}$, as follows [cf. Eq. (S41)]

$$H_{0\leftrightarrow 2} = \begin{pmatrix} 0 & J_1 & 0 & J_2 \\ J_1 & 2\omega_0 & J_2 & 0 \\ 0 & J_2 & 2\omega_0 & J_1 \\ J_2 & 0 & J_1 & 2\omega_0 \end{pmatrix}, \qquad H_{1\leftrightarrow 3} = \begin{pmatrix} \omega_0 & J_1 & 0 & J_2 \\ J_1 & \omega_0 & J_2 & 0 \\ 0 & J_2 & \omega_0 & J_1 \\ J_2 & 0 & J_1 & 3\omega_0 \end{pmatrix}. \tag{S58}$$

The full eigenfrequencies ω_n are plotted in Fig. S8 as the thin red lines, as a function of the dimerization parameter ϵ. The inadequacy of the RWA is most apparent in panel (c), where the coupling strength $\bar{J}$ is largest.

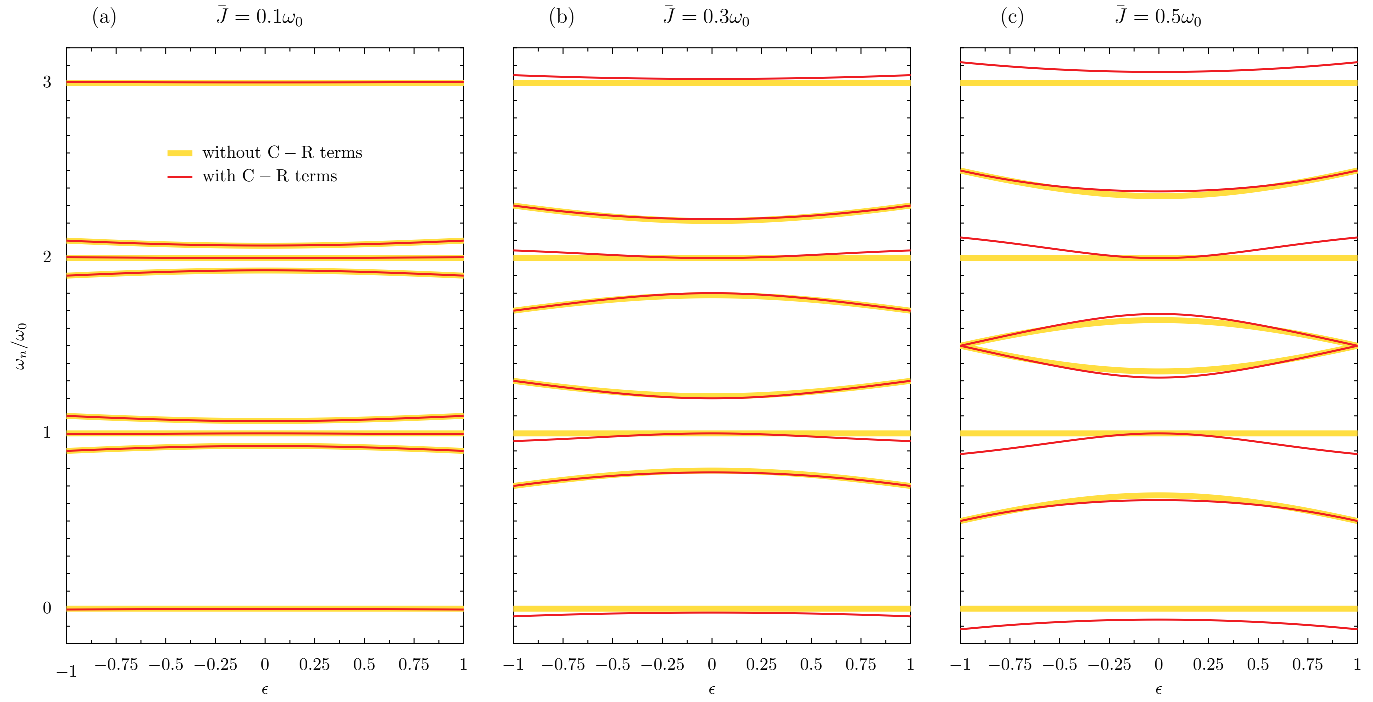

FIG. S8. **Eigenfrequencies in a dimerized triple of coupled two-level systems.** Eigenfrequencies in the strong (thick yellow lines) and ultrastrong (thin red lines) coupling regimes, as a function of the dimerization parameter ϵ. We consider the coupling strength $\bar{J}$ (in units of the transition frequency ω_0) to be (a) 0.1, (b) 0.3, and (c) 0.5.
